# Preventing traumatic stress–induced behavioral abnormalities in rats with blue light phototherapy

**DOI:** 10.1038/s41398-026-03981-z

**Published:** 2026-03-27

**Authors:** Yi Li, Weiwen Wang, Yizhou Tan, Haixia Qiu, Ying Wang, Jing Zeng, Defu Chen, Hongyou Zhao, Haolin Liu, Ying Gu

**Affiliations:** 1 Graduate School of PLA Medical College, Chinese PLA General Hospital and PLA Medical College, 28 Fu Xing Road, Beijing, 100083 China; 2 State Key Laboratory of Cognitive Science and Mental Health, Institute of Psychology, Chinese Academy of Sciences, Beijing, 100101 China; 3 Department of Laser Medicine, the First Medical Center of the PLA General Hospital, Beijing, China; 4 School of Medical Technology, Beijing Institute of Technology, Beijing, China; 5 No.965 Hospital, Joint Logistics Support Force of Chinese PLA, Jilin, China

**Keywords:** Learning and memory, Psychology

## Abstract

**Background:**

Posttraumatic stress disorder (PTSD) is a debilitating mental condition triggered by traumatic stress, and current treatments remain limited. Non-invasive phototherapy has shown promise in mood disorders, yet its efficacy in PTSD—particularly for early intervention and fear symptom mitigation—remains insufficiently explored.

**Objective:**

This study aimed to investigate the preventive and therapeutic effects of blue non-invasive phototherapy (470 nm) on PTSD-related anxiety and fear behaviors in a rat model, focusing on neuronal activation and transcriptomic changes in emotion-related brain regions.

**Methods:**

Sprague-Dawley (SD) rats (n = 100) were subjected to single inescapable electric foot shock after single prolonged stress procedure (SPS-S). Groups included control (Ctrl), SPS-S, SPS-S + immediate light therapy (SPS-S + I-LT), SPS-S + delayed light therapy (SPS-S + D-LT), and SPS-S + immediate and delayed light therapy combination (SPS-S + ID-LT). Behavioral assessments, including open field test (OFT), elevated plus maze (EPM) and fear conditioning, were conducted at week one and three post-intervention. Immunohistochemical staining for c-Fos expression and RNA sequencing of the medial prefrontal cortex (mPFC) were performed to evaluate neural activation and transcriptomic alterations.

**Results:**

I-LT and ID-LT were associated with reduced anxiety-like behaviors in the OFT and EPM compared with untreated SPS-S-exposed rats (p < 0.001). Improvements in fear-related freezing behavior were observed at 3 weeks in the I-LT group and ID-LT group (p < 0.001), whereas D-LT was associated with anxiolytic effects without significant changes in fear responses. SPS-S exposure was associated with increased c-Fos expression in the infralimbic mPFC, and this elevation was attenuated in animals receiving I-LT or ID-LT. Transcriptomic analyses revealed stress-associated alterations in synapse-related pathways, which were modulated in the I-LT group. Selected synaptic genes were further examined by quantitative real-time PCR.

**Conclusions:**

Blue light phototherapy produced distinct behavioral and molecular signatures in a rat model of traumatic stress. Immediate post-trauma intervention showed stronger associations with fear-related outcomes, supporting the potential relevance of early light-based interventions as a non-invasive adjunctive strategy for traumatic stress management.

## Introduction

Posttraumatic stress disorder (PTSD) is a psychiatric disorder that develops following exposure to traumatic events through direct experience or witnessing. Epidemiological evidence indicates that more than 70% of individuals are exposed to at least one traumatic event during their lifetime, though the lifetime prevalence of PTSD exhibits significant geographical variation ranging from 1.3–12.2% [1]. Clinical interventions mainly include pharmacological and psychological treatments. However, both of them have significant limitations. Thus, there is an urgent need for safe, effective, and non-toxic preventive and therapeutic approach for PTSD [2].

Since its application in 1984 for seasonal affective disorder, non-invasive phototherapy has gained popularity due to its non-invasive nature and effectiveness, gradually being applied to conditions such as non-seasonal depression and anxiety disorders [3–5]. Non-invasive phototherapy can alleviate negative emotions either by directly modulating activity in brain regions associated with the sympathetic nervous system or indirectly by regulating circadian rhythms and sleep [6]. Light, as a longstanding natural signal, plays a crucial role in regulating human circadian rhythms and emotional states, primarily mediated through the eye—the natural photoreceptive organ. Previous studies on blue non-invasive phototherapy (wavelength: 469 nm) have demonstrated significant effects in alleviating anxiety and improving sleep quality. For instance, Glickman et al. reported that blue light (398 lux) achieved similar clinical efficacy as bright white light (10,000 lux) in treating SAD [7], and Meesters et al. further reduced the blue light intensity to 100 lux, maintaining equivalent therapeutic efficacy while reducing adverse events such as eye fatigue and dizziness [8]. However, the role of blue non-invasive phototherapy in the prevention and treatment of PTSD remains unclear.

Considering that trauma-related stress disorders represent chronic psychiatric conditions, increasing evidence indicates that preventive interventions applied during a critical therapeutic window after stress exposure are of substantial clinical significance [9, 10]. A meta-analysis of 19 randomized controlled trials demonstrated that early hydrocortisone intervention post-trauma significantly improves PTSD symptoms and prognosis. [11] Consistent with this concept, our recent work showed that non-invasive phototherapy using 810 nm transcranial infrared light effectively prevented the development of PTSD-like symptoms, further supporting the feasibility of early intervention strategies [12].

Current researches on ocular non-invasive phototherapy for PTSD have primarily focused on long-term patients, demonstrating improvements in sleep disturbances and certain symptoms but limited efficacy in reducing fear symptoms. However, studies on its underlying mechanisms remain insufficient. For instance, one study found that blue light (469 nm, 214 lux) intervention significantly improved sleep disturbances and certain PTSD symptoms while increasing the left amygdala volume, as observed via MRI [13, 14]. In contrast, clinical trials using ultraviolet-filtered bright white light (without the blue light spectrum) showed no significant improvement in negative emotions such as anxiety and depression [15].

Stress exposure profoundly interferes with the body’s natural circadian rhythms and these circadian disturbances extend beyond the suprachiasmatic nucleus (SCN) to limbic and prefrontal regions involved in emotional regulation [16, 17]. The medial prefrontal cortex (mPFC) integrates both stress and circadian signals through dense reciprocal connections with the amygdala, hippocampus (HIP), and thalamus [18, 19]. Notably, the mPFC exhibits intrinsic diurnal fluctuations in neuronal excitability and c-Fos activation, which are disrupted under chronic stress conditions. Blue light phototherapy, through non-image-forming retinal pathways, can modulate these cortical oscillations and restore circadian alignment [20].

Collectively, although blue light non-invasive phototherapy has shown remarkable effects in improving anxiety and sleep disturbances, its efficacy in mitigating fear symptoms remains limited. Moreover, no studies to date have explored the preventive effects of blue non-invasive phototherapy in the early post-trauma period. Investigating the effects of early blue light non-invasive phototherapy on PTSD-related negative emotions may provide fundamental experimental evidence for the clinical and military applications of non-invasive phototherapy in the prevention and treatment of PTSD.

## Materials and methods

### Animals and experimental paradigm

**Fig. 1:**
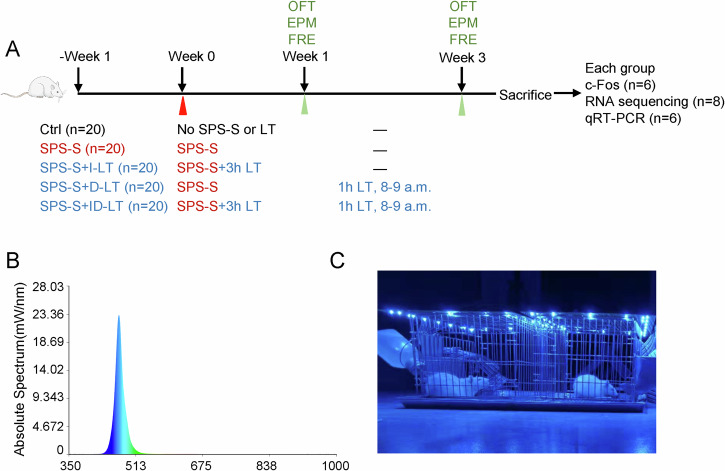
The detailed procedure for applying blue light therapy on rats. **A** Timeline of the experimental procedure. **B** The Absolute Spectrum (mW/nm) of blue light irradiation apparatus. **C** The illustrations of real-world scenarios during the blue light therapy procedure. Ctrl, control group; SPS-S, SPS-S group; SPS-S + I-LT, SPS-S + 3 h immediate light therapy group; SPS-S + D-LT, SPS-S + 1 h delayed blue light therapy for 14 days, 8–9 a.m. group; SPS-S + ID-LT, SPS-S + 3 h immediate light therapy + 1 h delayed blue light therapy for 14 days, 8–9 a.m. group.

Seven-week-old male Sprague–Dawley (SD) rats were purchased from SPF (Beijing) Biotechnology Co., Ltd, with an average body weight of approximately 240 g at arrival. Animals were singly housed in a specific pathogen–free (SPF) facility, in standard metal cages measuring 29 × 25 × 22 cm (length × width × height). The cages were suspended above corncob bedding, and animals had free access to food and water throughout the experiment. Animals were provided with standard rodent maintenance diet (SPF-F02-001) obtained from SPF (Beijing) Biotechnology Co., Ltd. This study randomly divided 100 seven-week-old Sprague-Dawley (SD) rats into five groups: control (Ctrl) group, single inescapable electric foot shock after single prolonged stress procedure group (SPS-S) group, SPS-S + immediate light therapy (SPS-S + I-LT) group, SPS-S + delayed light therapy (SPS-S + D-LT) group, and SPS-S + immediate and delayed light therapy combination (SPS-S + ID-LT) group, with 20 rats in each group. Following behavioral testing, randomly, 6 rats per group were used for immunohistochemical analysis, 8 rats per group were used for transcriptomic analysis of the infralimbic mPFC, and 6 rats per group were used for qRT-PCR analysis. Sample sizes were determined based on prior experience with similar SPS-S/light-therapy models and are consistent with commonly used group sizes. The experimental procedure is illustrated in Fig. 1A. Rats in the Ctrl group underwent neither modeling nor light therapy intervention. Specifically, all animals were maintained under a 12:12 h light/dark cycle (lights on at 07:00, lights off at 19:00) with controlled temperature (25 ± 1 °C) and humidity (50 ± 10%). The SPS-S group was subjected to the single inescapable electric foot shock after the single prolonged stress procedure to establish a PTSD-like model but did not receive non-invasive phototherapy. The SPS-S + I-LT group was subjected to the SPS-S group method to establish the PTSD-like model, followed by a 30 min rest and then immediate light therapy, specifically 3 h of non-invasive phototherapy using a 470 nm, 500 lux blue LED (as shown in Fig. 1B, C). The D-LT group received delayed light therapy, starting on the 10th day after modeling, with daily interventions from 8:00–9:00 AM for 14 consecutive days. The ID-LT group received a combination of immediate and delayed light therapy, constituting full-cycle light therapy. The animal experimental protocol was approved by the Institutional Animal Care and Use Committee (IACUC) of Chinese People’s Liberation Army (PLA) General Hospital, and all the procedures were carried out in accordance with the approved guidelines.

### Construction of SPS-S model

To induce PTSD-like symptoms, the rats were subjected to a multi-stressor protocol adapted from previous studies [12, 21]. This protocol involved restraining the rats in a plastic container for 2 h, followed by a 20 min forced swim in water maintained at 24 ± 1 °C with a depth of 50 cm. The rats were then allowed a 15 min rest period. After resting, the rats were anesthetized until they were unconscious and then administered 10 random electric shocks at an intensity of 1 mA, each lasting 6 s, over a 15 min interval. After these stressors, the rats were returned to their cages with sufficient food and water for a week before undergoing behavioral assessments.

### Apparatus and phototherapy

The light source for irradiation was a blue LED light panel (Yeolight Tech Co., Beijing, China). The panel measures 31 × 27 cm, with an effective illumination area of 29 × 25 cm, which match the side length of a single housing cage (height: 22 cm)(Supplementary Fig 1). The panel was positioned 22 cm above the cage, at which distance the illumination uniformity exceeded 90%. The illuminance was set to 500 lux (approximately 3.5 mW/cm²) and was measured using a SPIC-301 spectrocolorimeter. The system maintained constant temperature (25 ± 1 °C) to avoid thermal effects. Detailed blue light parameters are shown in Supplementary Table 1, and the phototherapy procedure is illustrated in Fig. 1C. During the intervention, rats were allowed to move freely, and theoretically, there would be no significant differences in light exposure levels among the animals.

### Open field test (OFT)

Following a 60 min period of acclimation under low light conditions (15 lux), the rats were introduced into the center of a plastic arena, where they were permitted to explore freely for 10 min. Their movements were tracked and recorded by an overhead infrared camera. The open field apparatus consisted of a square arena measuring 100 × 100 × 50 cm (length × width × height). The central zone was defined using Any-Maze software by dividing the entire arena into a 5 × 5 grid (25 equal squares), with the central 3 × 3 squares designated as the center area for analysis. The frequency of entries of the center area, time spent in the center area and distances traveled in center area were quantified and analyzed using Any-Maze software.

### Elevated plus maze (EPM) test

After a 60 min adjustment period in a dimly lit room, the rats were positioned on the central platform of the maze, oriented towards the same open arm, and were given 10 min to explore. The elevated plus maze comprised two open arms and two closed arms arranged perpendicularly in a cross-shaped layout and mounted on a platform 50 cm above the ground. The open arms were 42.5 cm long and 12 cm wide, with a 2 cm rim, while the closed arms had identical dimensions but were surrounded by 22.5 cm-high walls. The open arms were more brightly illuminated than the closed arms to increase their aversiveness, as commonly employed in elevated plus maze testing. An infrared camera above the maze tracked their behavior, recording the number of entries of both the open and closed arms, the time spent in both the open and closed arms and the distances traveled in both the open and closed arms. These data were analyzed with Any-Maze software, and the percentage of entries/time/distance in the open arm was calculated as: (entries/time/distance in open arms)/[(entries/time/distance in open arms) + (entries/time/distance in closed arms)] * 100%.

### Fear conditioning test

Fear-related behavior was assessed using a contextual fear test. Rats were re-exposed to the same shock chamber used during the stress induction phase, and freezing behavior was continuously recorded over a 6-min testing period with no stimulation. The cumulative duration of immobility was used as a measure of fear level. Freeze Frame software was utilized to measure and analyze the freezing time for the entire period. The freezing percentage was calculated to assess the rats’ level of fear, with the data plotted accordingly.

### Immunohistochemical staining

At 1.5 h after the final behavioral test, rats were deeply anesthetized with sodium pentobarbital (50 mg/kg, i.p.) and transcardially perfused with 0.9% saline followed by 4% paraformaldehyde (PFA). Brains were post-fixed overnight at 4 °C and subsequently transferred to 30% sucrose for cryoprotection. Coronal brain sections (40 μm thickness) were cut using a cryostat (Leica CM1950). Sections containing mPFC (cingulate cortex area 1, Cg1; prelimbic cortex, PrL; infralimbic cortex, IL) and HIP (cornu ammonis 3, CA3; dentate gyrus, DG) were selected for c-Fos immunofluorescence staining. For each rat, three serial sections spaced 120 μm apart were analyzed for each region. Free-floating sections were blocked in 5% normal goat serum containing 0.3% Triton X-100 for 1 h at room temperature and then incubated overnight at 4 °C with a rabbit anti-c-Fos antibody (1:500, Synaptic Systems, #226003). After washing, sections were incubated with Alexa Fluor 488–conjugated goat anti-rabbit IgG (1:500, Abcam) for 1 h at room temperature and counterstained with DAPI. Images were acquired under identical settings using a Zeiss Axio Imager M2 microscope. Regions of interest were defined according to the Paxinos and Watson rat brain atlas. Quantification of c-Fos–positive nuclei was performed using ImageJ after thresholding, and the mean number of positive cells per mm² was calculated from three sections per rat. All imaging and analysis parameters were kept constant across groups.

### RNA sequencing and analysis

For transcriptional analysis, brain tissue was rapidly frozen and sectioned in a cryostat, and the infralimbic mPFC region was accurately sampled using a punch needle. Total RNA of mPFC (IL) was isolated using RNAprep Pure Tissue Kit (TIANGEN, DP431) and assessed for quality (RIN ≥ 6.5, OD260/280 = 1.8–2.2, 28S:18S ≥ 1.0) using an Agilent 5300 Bioanalyzer and NanoDrop ND-2000 spectrophotometer. Only high-quality RNA ( ≥ 1 μg) was used for library preparation. Stranded mRNA libraries were prepared from poly-A-enriched RNA (1 μg input) using the Illumina® Stranded mRNA Prep kit, including fragmentation, double-stranded cDNA synthesis (SuperScript kit, Invitrogen), end repair, adapter ligation, and size selection (300 bp fragments). PCR amplification was performed with Phusion DNA polymerase (15 cycles). Libraries were sequenced on the NovaSeq X Plus (Illumina, PE150) or DNBSEQ-T7 (MGI, PE150) platforms.Raw reads were quality-trimmed using fastp and aligned to the reference genome (HISAT2). Transcript assembly was performed with StringTie. Gene expression was quantified as TPM using RSEM. Differentially expressed genes (DEGs) were identified with DESeq2 (|logFC | ≥ 1, P.Val < 0.05). Gene Ontology (GO) and Genes and Genomes (KEGG) pathway analyses were conducted using Goatools and SciPy, respectively, with significance thresholds (P < 0.05). Splice variants (exon skipping, alternative 5′/3′ sites, intron retention) were detected using rMATS.

### Quantitative real-time PCR (qRT-PCR) analysis

Total RNA was isolated from mPFC (IL) using TRIzol reagent (Life Technologies, USA). Briefly, tissues were homogenized in TRIzol, followed by chloroform phase separation and isopropanol precipitation. RNA pellets were washed with 75% ethanol, air-dried, and dissolved in DEPC-treated water. RNA concentration and purity were determined via NanoDrop spectrophotometry. cDNA synthesis was performed using 500 ng RNA with M-MLV Reverse Transcriptase (Promega, USA) and random primers under standard conditions (70 °C for 5 min; 37 °C for 60 min). SYBR Green-based qPCR (CWBIO, China) was conducted in 25 μL reactions containing 2 × SYBR Mixture, gene-specific primers (designed via PerlPrimer and synthesized by Beijing Genomics Institution, China; sequences in Supplementary Table 3), and cDNA. Amplification was performed on a thermal cycler with the following protocol: 95 °C for 10 min; 40 cycles of 95 °C/15 s and 60 °C/1 min. GAPDH served as the endogenous control, with technical duplicates per sample. Cycle threshold (Ct) values were analyzed using the 2 − ΔΔCt method.

### Retinal hematoxylin–eosin (HE) staining

To determine whether blue light exposure caused retinal damage in rats, we conducted a separate ID-LT and compared the outcomes with those of untreated control animals. Eyes from rats in both groups were collected immediately after humanely euthanized following anesthesia. The enucleated eyeballs were fixed in 4% paraformaldehyde for 24 h, followed by standard paraffin embedding and sectioning. After deparaffinization and hematoxylin–eosin (HE) staining, retinal sections were imaged under a 40 × objective. The outer nuclear layer (ONL) thickness was quantified using Case Viewer software. Measurements were performed at two locations situated 2 mm to the left and right of the optic disc. Each experimental group included eight rats, and both eyes from each animal were analyzed, with measurements obtained separately from the left and right retina.

### Statistical analysis

All datasets were tested for normality (Shapiro–Wilk test) and homogeneity of variance (Levene’s test). One-way ANOVA was used to analyze differences among groups, followed by Tukey’s HSD post hoc tests as appropriate. Also, data were analyzed using two-way mixed ANOVA with Time (within-subject: Week 1, Week 3) and Treatment (between-subject: Ctrl, SPS-S, I-LT, D-LT, ID-LT) as factors. Data are expressed as mean ± SEM, and statistical significance was defined as P < 0.05 (two-tailed). Behavioral assessments and outcome analyses were performed by investigators blinded to the treatment groups. Statistical analyses were performed using GraphPad Prism 10 (GraphPad Software, USA) and SPSS 26.0 (IBM Corp., USA).

## Results

### 470 nm light irradiation apparatus developed for the treatment of SPS-S rats

An LED light panel with a central wavelength of 470 nm was used for light therapy in freely moving rats, with an illuminance of 500 lux. The size of the light panel is 31 × 17 cm, and the actual illuminated area is 29 × 25 cm (Supplementary Fig. 1A), equipped with 30 LED bulbs (Supplementary Fig. 1B). In a darkroom environment, the light panel was fixed 22 cm above the measurement point (Supplementary Fig. 1C). Illuminance was measured at the top-left, top-center, top-right, middle-left, center, middle-right, bottom-left, bottom-center, bottom-right, and two random points. The final measurement results showed a uniformity of over 90% (Supplementary Fig. 1D).

### I-LT and ID-LT prevented PTSD-like anxiety in OFT and EPM (assessed at week 1)

To investigate the effect of blue light therapy on anxiety-like behavior, the OFT and EPM were performed [22, 23].

**Fig. 2:**
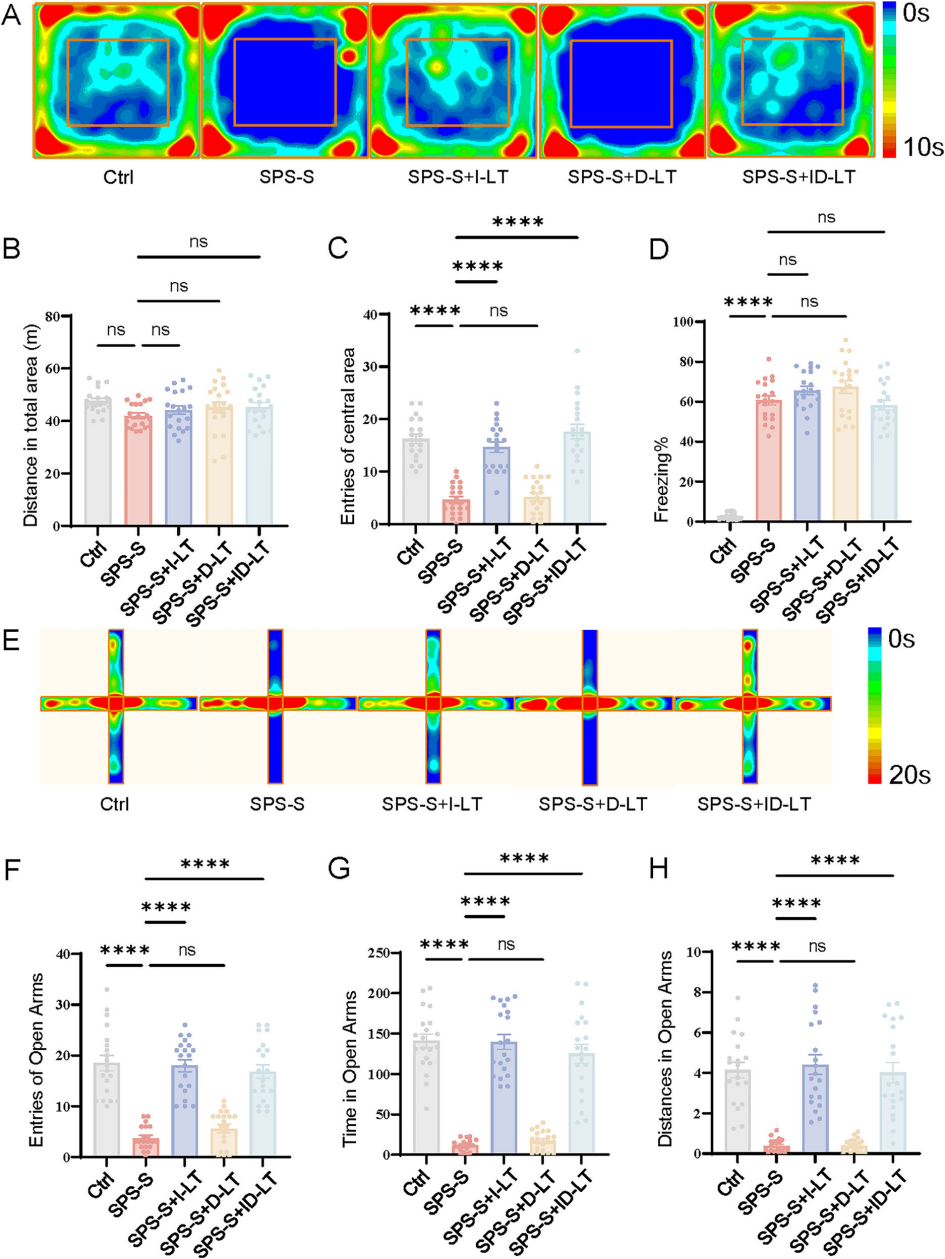
Behavioral assessments at Week 1 following SPS-S exposure. **A** Heatmaps of time spent in open field test (OFT). **B** Total distance moved in OFT (**C**) Entries into central area in OFT. **D** The percentage of freezing time in 6 mins. **E** Heatmaps of time spent in elevated plus maze (EPM). **F** Entries into open arms in EPM. **G** Time spent in open arms in EPM. **H** Distance moved in open arms in EPM. Data are presented as mean ± SEM. *P < 0.05, **P < 0.01, ***P < 0.001, ns, no statistical difference. Each group, n = 20, one-way ANOVA.

In the OFT, the number of entries into the central area was significantly reduced in the SPS-S group compared to the Ctrl group (4.60 ± 2.89 vs. 16.20 ± 3.87, P < 0.001, Fig. 2A, C), indicating elevated anxiety levels. Increased center entries in the SPS-S + I-LT group (14.65 ± 4.50) and the SPS-S + ID-LT group (17.60 ± 6.18), which were comparable to the Ctrl group (all P > 0.05 vs. Ctrl, Fig. 2C). At this stage, the SPS-S + D-LT group had not yet received any light-therapy intervention, and therefore showed no significant difference compared with the SPS-S group (P = 0.9944, Fig. 2C).

To exclude potential locomotor confounds, total distance traveled was analyzed and showed no significant difference among the five groups (F (4, 95) = 1.771, P = 0.14; Fig. 2B), confirming that the observed behavioral effects were not due to altered motor activity.

In the EPM, the SPS-S group exhibited markedly reduced open-arm exploration compared with the Ctrl, including fewer open-arm entries (3.75 ± 2.34 vs. 18.50 ± 6.74), shorter time spent in the open arms (11.72 ± 6.88 s vs. 140.91 ± 37.88 s), and shorter open-arm distance traveled (0.39 ± 0.32 m vs. 4.14 ± 1.70 m; all *P* < 0.001; Fig. 2E-H), consistent with heightened anxiety levels. I-LT significantly improved these measures, with both the SPS-S + I-LT group and the SPS-S + ID-LT group showing enhanced open-arm exploration that closely approached control levels (e.g., time spent: 139.77 ± 40.99 s and 125.08 ± 51.86 s, respectively; *P* < 0.001 vs. SPS-S; Fig. 2G) In contrast, the SPS-S + D-LT group, which had not yet undergone light therapy at this point, exhibited no behavioral recovery (*P* > 0.50; Fig. 2E-H).

### I-LT and ID-LT prevented PTSD-like anxiety in OFT and EPM (assessed at week 3)

Over the following two weeks, the SPS-S + D-LT group and the SPS-S + ID-LT group received one hour of blue light therapy per day, whereas the SPS-S + I-LT group received no further treatment.

**Fig. 3:**
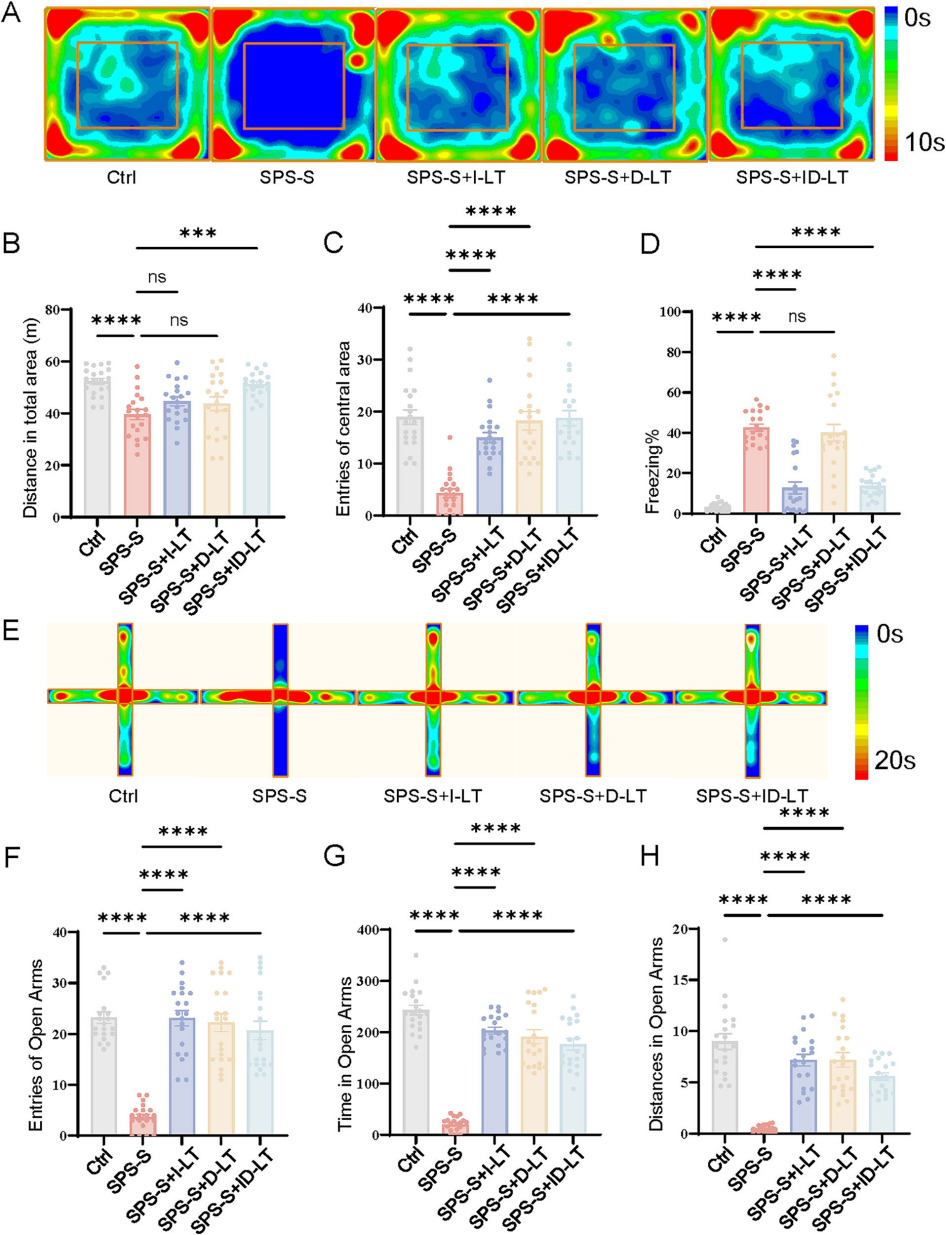
The behaviors of rats in at Week 3 following SPS-S exposure. **A** Heatmaps of time spent in open field test (OFT). **B** Total distance moved in OFT (**C**). Entries into central area in OFT. **D** The percentage of freezing time in 6 mins. **E** Heatmaps of time spent in elevated plus maze (EPM). **F** Entries into open arms in EPM. **G** Time spent in open arms in EPM. **H** Distance moved in open arms in EPM. Data are presented as mean ± SEM. *P < 0.05, **P < 0.01, ***P < 0.001, ns, no statistical difference. Each group, n = 20, one-way ANOVA.

In OFT, compared with the SPS-S group, the SPS-S + D-LT group showed significantly increased entries into the central zone (18.20 ± 7.96 vs. 4.25 ± 3.73, P < 0.001, Fig. 3A-C). The SPS-S + I-LT group maintained a low anxiety state without further improvement, and no additive therapeutic effect was observed in the SPS-S + ID-LT group.

Consistent results were obtained in EPM. The SPS-S + D-LT group exhibited significantly increased open-arm exploration compared with the SPS-S group, including higher open-arm entries (22.25 ± 7.85 vs. 3.70 ± 2.49), longer open-arm duration (191.64 ± 60.35 s vs. 20.32 ± 13.68 s), and greater open-arm movement distance (7.21 ± 3.18 m vs. 0.45 ± 0.35 m; all P < 0.001; Fig. 3E-H). The SPS-S + I-LT group also showed a sustained alleviation of anxiety symptoms, yet combined intervention failed to produce any synergistic enhancement.

Two-way mixed ANOVA further demonstrated robust Time × Treatment interactions for both OFT and EPM measures (all *P* < 0.0001), confirming that the behavioral improvements over time differed significantly across treatment groups. Post hoc tests revealed that D-LT produced delayed anxiolysis, whereas I-LT maintained early improvements. Total distance remained unaffected by treatment (no significant Time effect), confirming that anxiety-related outcomes were not confounded by motor activity (Supplementary Table 4, Supplementary Fig. 2A-E).

### I-LT and ID-LT but not D-LT prevented PTSD-like fear in Freezing test

The dysregulation of fear-related memory consolidation is widely recognized as a central pathophysiological hallmark of PTSD [24]. At week 1 following I-LT, no measurable therapeutic effect was observed. The SPS-S group displayed markedly elevated freezing behavior compared with controls (60.64 ± 9.98% vs. 2.69 ± 1.71%, P < 0.001; Fig. 2D), indicative of persistent fear memory consolidation. Neither the SPS-S + I-LT group (65.65 ± 9.51%, P = 0.49) nor the SPS-S + ID-LT group (58.27 ± 10.86%, P = 0.96) showed significant improvement relative to the SPS-S group. Similarly, the D-LT—prior to illumination—exhibited no observable difference (67.29 ± 13.81% vs. 60.64 ± 9.98%, P = 0.42).

After a subsequent two-week D-LT intervention, freezing levels remained unchanged, showing no difference from untreated SPS-S rats (40.02 ± 18.76% vs. 42.64 ± 7.88%, P = 0.98; Fig. 3D). In contrast, by three weeks post-intervention, both the SPS-S + I-LT group (12.69 ± 13.33%, P < 0.001) and the SPS-S + ID-LT group (13.70 ± 5.81%, P < 0.001) groups exhibited a robust attenuation of freezing behavior compared with the SPS-S group (42.64 ± 7.88%).

Two-way mixed ANOVA showed a highly significant Time × Treatment interaction (F (4,95) = 39.77, P < 0.0001), along with significant main effects of Time (F (1,95) = 356.8, P < 0.0001) and Treatment (F (4,95) = 147.1, P < 0.0001). Post hoc analyses confirmed that the I-LT group and ID-LT group showed strong decreases in freezing from Week 1 to Week 3, whereas the D-LT group showed no such improvement. (Supplementary Table 4, Supplementary Fig. 2F).

Collectively, these results indicate that I-LT—but not D-LT—effectively prevents maladaptive fear consolidation and facilitates fear extinction, emphasizing the importance of timely intervention immediately after trauma exposure.

### HE staining results

To evaluate whether blue-light exposure induced retinal toxicity, we examined retinal morphology following the full course of ID-LT. HE staining was performed on paraffin-embedded retinal sections collected after ID-LT, and retinal thickness was quantitatively analyzed at standardized locations (2 mm nasal and temporal to the optic disc). No gross structural abnormalities or retinal layer disorganization were observed in ID-LT rats compared with untreated controls. Quantitative analysis further confirmed the absence of retinal damage, as ONL did not differ between groups (Ctrl: 37.05 ± SEM μm; ID-LT: 36.14 ± SEM μm; unpaired t-test: t(14) = 0.67, P = 0.51; Supplementary Fig. 3). These findings indicate that the illumination parameters used in this study did not elicit detectable histological alterations in the retina.

### I-LT and ID-LT but not D-LT prevented the upregulation of expression of c-Fos in the mPFC

**Fig. 4:**
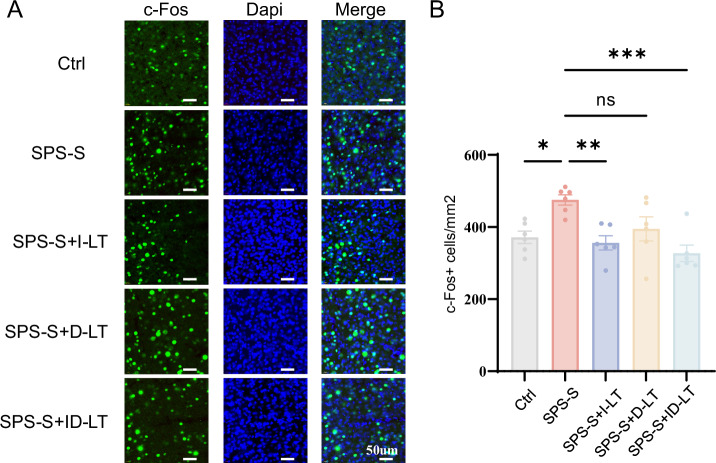
The effect of phototherapy on c-Fos expression in infralimbic cortex (IL). **A** Schematic diagram of immunofluorescence results in the IL region. **B** Statistical diagram of c-Fos density results in the IL region. *P < 0.05, **P < 0.01, ***P < 0.001, ns, no statistical difference. Each group, n = 6, one-way ANOVA.

Emerging evidence indicates that aberrant neuronal activity within mPFC and HIP represents a core neurobiological feature of posttraumatic stress–related psychopathology, with c-Fos expression widely used as a marker of stimulus-evoked neuronal activation [25, 26]. Schematic representations of the immunofluorescence sampling regions in the mPFC (Cg1, PrL, IL) and HIP (CA3, DG) are shown in Supplementary Fig. 4.The Supplementary Figs. 5–8 indicated that no significant differences in c-Fos expression were observed among groups in Cg1 (F (4,25) = 2.276, P = 0.089), Prl (F (4,25) = 1.429, P = 0.254), CA3 (F (4,25) = 1.565, P = 0.214) and DG (F (4,25) = 1.429, P = 0.254). Significant group differences were detected in IL (F (4,25) = 6.267, P = 0.001). The SPS-S group exhibited elevated c-Fos levels vs. Ctrl (475.10 ± 35.00 vs. 371.42 ± 42.19, P = 0.024). I-LT (I-LT group: 355.91 ± 48.30, P = 0.007; SPS-S + ID-LT group: 327.23 ± 55.27, P < 0.001) but not D-LT (D-LT group: 394.69 ± 81.62, P = 0.114) normalized SPS-S-induced elevation (Fig. 4A, B). Collectively, these findings indicate that I-LT, either alone or combined with delayed treatment, was associated with an attenuation of SPS-S–related increases in IL mPFC neuronal activation, while D-LT alone showed no detectable effect at this level.

### Gene ontology and Kegg analysis of differentially expressed genes

**Fig. 5:**
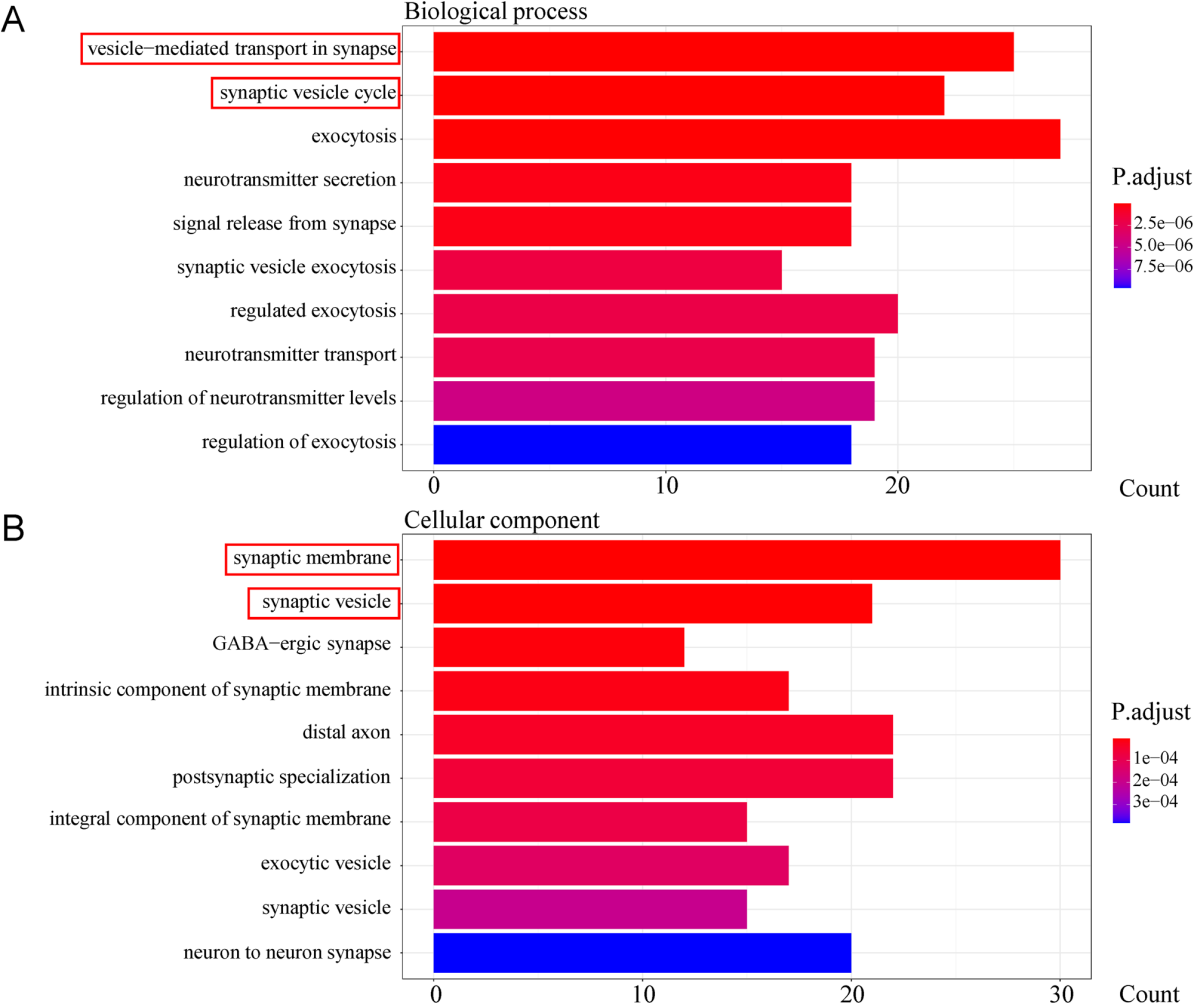
GO enrichment analysis of upregulated differentially expressed genes (DEGs) in the SPS-S group compared with the Ctrl group. **A** Top 10 enriched biological process pathways. **B** Top 10 enriched cellular component pathways. *DEGs were identified by RNA sequencing of tissue punches collected from the infralimbic mPFC (IL).

**Fig. 6:**
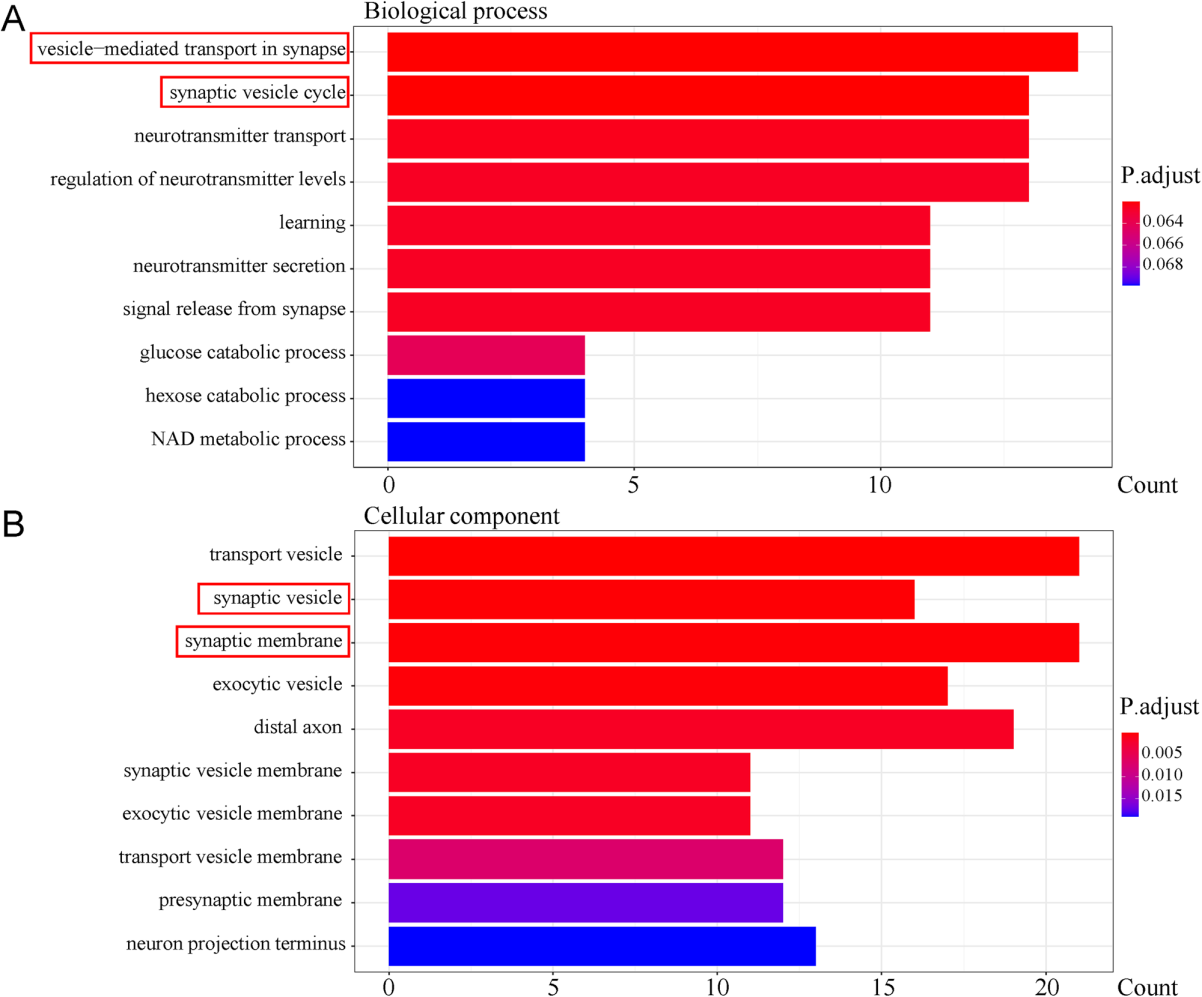
GO enrichment analysis of downregulated DEGs in the I-LT group compared with the SPS-S group. **A** Top 10 enriched biological process pathways. **B** Top 10 enriched cellular component pathways. *DEGs were identified by RNA sequencing of tissue punches collected from the infralimbic mPFC (IL).

Differential expression analysis (screening criteria: P.Val<0.05, |logFC | >1) identified 434 DEGs between the SPS-S and the Ctrl groups (203 upregulated, 231 downregulated; Supplementary Fig. 9A) and 653 DEGs between the SPS-S + I-LT group and the SPS-S group (305 upregulated, 348 downregulated; Supplementary Fig. 9B). GO enrichment revealed reciprocal pathway patterns: SPS-S-upregulated DEGs (vs Ctrl) were enriched in synaptic vesicle-related processes (vesicle-mediated synaptic transport, synaptic vesicle cycle; synaptic membrane and vesicle). Strikingly, I-LT-downregulated DEGs (vs SPS-S) mirrored SPS-S-upregulated patterns in synaptic pathways (synaptic vesicle transport/cycle in BP; synaptic vesicles), while I-LT-upregulated DEGs exhibited EC-M-related enrichments overlapping with SPS-S-downregulated terms (Figs. 5, 6; Supplementary Figs. 10–11).

**Fig. 7:**
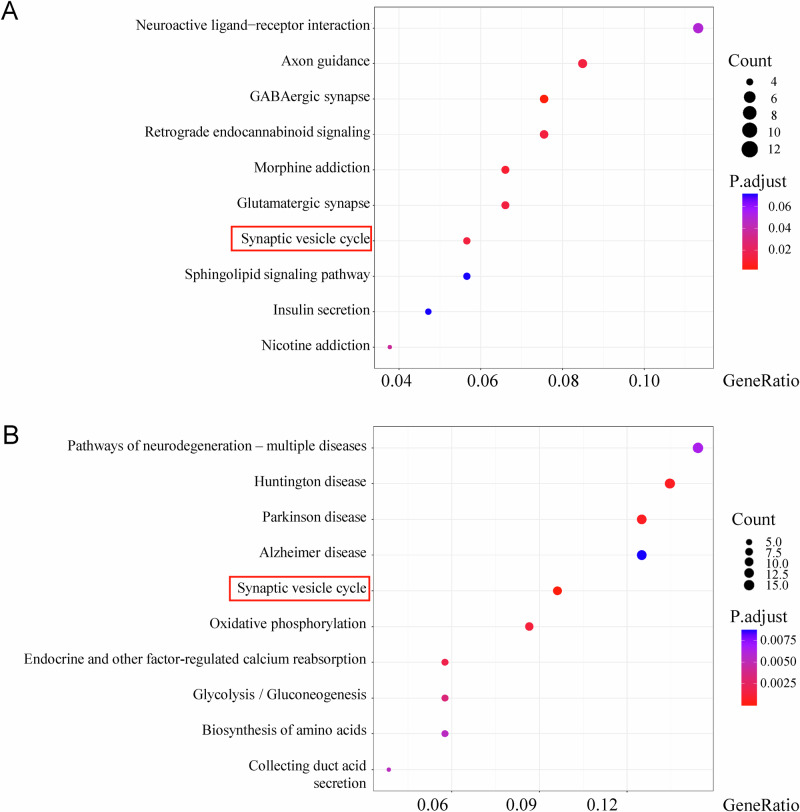
KEGG enrichment analysis of upregulated differentially expressed genes (DEGs) in the SPS-S group compared with the Ctrl group and downregulated DEGs in the I-LT group compared with the SPS-S group. **A** Top 10 significantly enriched pathways of upregulated DEGs. **B** Top 10 significantly enriched pathways of downregulated DEGs. *DEGs were identified by RNA sequencing of tissue punches collected from the infralimbic mPFC (IL).

KEGG analysis confirmed pathway concordance: synaptic vesicle cycle was enriched in both SPS-S-upregulated DEGs (vs Ctrl) and I-LT-downregulated DEGs (vs SPS-S) (Fig. 7). These findings demonstrate systematic reversal of synaptic and ECM pathway alterations between the SPS-S group and the SPS-S + I-LT group at both GO and KEGG levels.

### qRT-PCR Validation of representative differentially expressed genes

To validate the RNA Sequencing results, we performed qRT-PCR analysis on nine representative genes exhibiting significant p-value differences to confirm their mRNA-level alterations. In the mPFC region, mRNA expression analysis of the nine differentially expressed genes revealed significant intergroup variations in Bhmt (F (2, 15) = 52.89, p < 0.001, Supplementary Fig. 12A), Syt6(F (2, 15) = 6.094, p = 0.0116, Supplementary Fig. 12B), and Slc17a6(F (2, 15) = 8.683, p = 0.0031, Supplementary Fig. 12C), with the SPS-S group showing higher or lower mRNA levels compared to the Ctrl group and the SPS-S + I-LT group. The remaining six genes (Napb, Grm8, Mat2b, Amph, Apba2, and Snap25) demonstrated no statistically significant differences across experimental groups (Supplementary Fig. 12D–I). These validation results support the reliability of the sequencing data.

## Discussion

Early blue light therapy (470 nm) was associated with marked improvements in PTSD-like anxiety and facilitated fear extinction in rats, particularly when administered immediately after trauma or combined with delayed intervention. At the neural level, these behavioral effects were accompanied by an attenuation of mPFC hyperactivity and by transcriptional changes in synapse-related pathways involving genes such as Bhmt and Syt6. Collectively, these findings suggest that blue light therapy may exert its most pronounced behavioral and molecular associations when delivered within an early post-trauma window, supporting its potential as a non-invasive, drug-free approach for early PTSD intervention.

These results suggest that light therapy has emerged as a safe and effective modality in clinical practice, having been established as a first-line treatment for seasonal affective disorder [27]. Moreover, it has demonstrated favorable therapeutic effects for non-seasonal depression [28], anxiety [5], premenstrual syndrome [29], and other mood disorders. I-LT can attenuate anxiety symptoms and facilitate fear extinction, an effect that may be associated with the “golden six-hour” window following trauma. Traumatic fear memories are consolidated via protein synthesis [30]. Previous studies have demonstrated that pharmacological agents such as hydrocortisone, which transiently enhance the hypothalamic–pituitary–adrenal axis response and modulate central protein synthesis, have been shown to effectively prevent stress-related symptoms at six months post-trauma, thereby improving quality of life [11, 31]. In parallel, non-invasive phototherapy exerts immediate effects on emotion-regulating brain regions through non-image-forming pathways mediated by intrinsically photosensitive retinal ganglion cells [32].

In contrast, D-LT was associated with improvements in anxiety-like behaviors but did not significantly alter fear-related responses. This dissociation is consistent with clinical observations from morning light therapy trials, in which improvements in mood and anxiety are frequently reported without robust effects on fear memory per se [33]. The behavioral effects observed with D-LT may therefore reflect indirect mechanisms, including normalization of circadian rhythms and improvements in sleep quality. Restoration of circadian rhythmicity and sleep architecture has been shown to alleviate anxiety symptoms in SPS-S model and clinical populations [34], and such processes may contribute to the anxiolytic effects observed in the delayed intervention paradigm. Together, these findings suggest that distinct light therapy schedules may differentially engage behavioral domains relevant to PTSD, depending on timing relative to trauma exposure.

C-Fos is a prototypical immediate-early gene and is widely used as an indicator of neuronal activation in response to external stimuli [35, 36]. In the classical single prolonged stress model, structural and functional alterations have been reported across multiple brain regions implicated with traumatic stress exposure, including the mPFC, HIP, and amygdala [37]. Previous studies indicate that c-Fos expression in the mPFC may vary dynamically over time following stress exposure, with early reductions in activity followed by later-stage hyperactivation in specific subregions such as the prelimbic and infralimbic cortices [38, 39]. Notably, experimental suppression of mPFC hyperactivity has been shown to ameliorate abnormal electroencephalographic patterns and sleep disturbances in SPS-based models [40], supporting a link between prefrontal dysregulation and PTSD-related phenotypes. In the present study, SPS-S exposure was associated with elevated c-Fos expression specifically in the IL region of the mPFC, while I-LT was associated with a reduction of this elevation toward levels observed in control animals. Although these findings are consistent with the notion that modulation of prefrontal activity may accompany behavioral improvement, the absence of a non–SPS-S light therapy control group precludes definitive separation of stress-specific reversal effects from potential stress-independent influences of light exposure on neuronal activation. Accordingly, the observed molecular changes should be interpreted as associative rather than strictly causal, and future studies incorporating additional control conditions will be required to more precisely delineate the specificity of light-induced neural modulation. Neuroimaging studies in PTSD patients, primarily using functional magnetic resonance imaging (fMRI), have similarly identified aberrant activation patterns and structural alterations within the mPFC and HIP [41–43]. The convergence between these clinical observations and the region-specific alterations detected in the present animal model underscores the translational relevance of the mPFC, particularly the IL subregion, in fear extinction and PTSD pathophysiology.

From a safety perspective, blue-light exposure has raised concerns primarily at short wavelengths (400–460 nm) and at irradiance levels exceeding 10 mW/cm² [44]. In contrast, the illumination parameters used in the present study (470 nm, 500 lux, approximately 3.5 mW/cm²) fall well below established phototoxicity thresholds and comply with international safety standards (IEC/EN 62471; ICNIRP, 2013). Consistent with the biphasic dose–response characteristics of photobiomodulation, the low-irradiance exposure employed here is expected to reside within a neuromodulatory, rather than phototoxic, range.

Based on the behavioral outcomes and the associated c-Fos findings, the I-LT paradigm was selected for transcriptomic analysis. The observed bidirectional regulation of synapse-related pathways aligns with prior reports of early stress–induced synaptic remodeling within the prefrontal cortex in SPS-S model [45]. In clinical settings, transcriptomic studies of PTSD have largely relied on peripheral blood samples [46] or post-mortem brain tissue [47]. Integrating region-specific transcriptomic profiling in animal models with human datasets may therefore provide valuable insights into conserved molecular signatures underlying PTSD pathogenesis [48].

Regarding the selection of intervention parameters, the I-LT protocol was designed based on the six-hour window for fear memory consolidation post-trauma and the clinical concept of a “golden six hours” [30]. The “golden six hours” window refers to the early post-trauma period when fear-memory consolidation is still labile. Prior basic and translational research shows that interventions delivered during consolidation or reconsolidation can produce enduring fear reduction, with extinction training during the reconsolidation window yielding persistent attenuation of fear in rodents and humans [47, 49, 50]. Clinical evidence similarly indicates that post-trauma timing is mechanistically critical: early pharmacological interventions—particularly hydrocortisone administered within hours—reduce subsequent PTSD symptoms and are supported by RCTs and meta-analyses [51, 52]. Early behavioral interventions in acute settings show comparable benefits. Additionally, morning blue-light protocols improve sleep, alleviate traumatic-stress symptoms, and enhance extinction-related processes in PTSD populations, including amygdala modulation. Together, these findings implicate a time-sensitive therapeutic window and support integrating blue-light intervention with existing early psycho- or pharmacological strategies [53–55]. Accordingly, the protocol involved a 30 min rest period following the traumatic event, succeeded by a 3 h light therapy session. To minimize potential disruptions to the circadian system, the duration of light therapy was confined within the light phase, and a uniform 3 h intervention period was adopted to control for pre- and post-intervention temporal variability. For the D-LT protocol, SD rats, which are nocturnal, were utilized; previous studies have indicated that nocturnal light exposure can induce depressive-like behaviors in these animals, whereas daytime exposure does not [56]. Consequently, a 1 h blue light intervention was administered each morning. In clinical trials, transcranial light therapy using blue light-enhanced white light has demonstrated effects comparable to those of bright white light and superior outcomes relative to red light controls in ameliorating negative affect [57].

In summary, this study demonstrates that temporally targeted blue light therapy is associated with differential behavioral and molecular signatures in a SPS-S rat model of PTSD-like symptoms. As a non-invasive, drug-free physical intervention, blue light therapy may represent a promising adjunctive strategy for early PTSD management. Future studies incorporating additional control conditions, mechanistic manipulations, and diverse experimental models will be essential to further elucidate the specificity and causal pathways underlying light-based neuromodulation in PTSD.

## Limitations

In addition, although the selected blue light parameters (470 nm, 500 lux, 3 h) were based on previous non-invasive phototherapy studies, the gradient effects of different light intensities or exposure durations were not investigated in the current work. Future studies should include a systematic parameter optimization to determine the most effective and safe light dosage for PTSD-like symptoms prevention. Regarding the examination of regional brain functional activity, the limitations of the experimental equipment precluded the use of confocal microscopy for layered cell counting; instead, a conventional microscope was used for c-Fos counting, which may have affected the accuracy of the counts. While the behavioral and molecular outcomes observed in this study strongly support the efficacy of blue-light light therapy, the absence of a sham-light comparison precludes firm conclusions about wavelength specificity. Prior studies have shown that blue light (~470 nm) exerts stronger effects on emotional regulation and circadian signaling than longer wavelengths [32, 34, 58, 59], suggesting a plausible mechanistic basis for our findings, yet further controlled studies are warranted. A limitation of this study is the absence of a sham light control group. Future experiments should incorporate an illumination control using long-wavelength red light, which is minimally detected by the murine visual system, to better differentiate wavelength-specific effects from general illumination exposure. Moreover, due to individual experimental cycle constraints, a comprehensive analysis of functional activity in other emotion-related brain regions, such as the amygdala and habenula, was not conducted. In the transcriptomic analysis, the validation method was confined to RNA-level confirmation via RT-PCR, without further protein-level validation using techniques such as Western Blot.

## Supplementary information

Supplement Table

Supplement Figure

## Data Availability

The data supporting the findings of this study are available within the article and its Supplementary Information files. Additional raw data are available from the corresponding author upon reasonable request.
