# Supplementary material for: Preventing traumatic stress–induced behavioral abnormalities in rats with blue light phototherapy
Source: Transl Psychiatry. 2026 Mar 27;16:211. doi: 10.1038/s41398-026-03981-z (PMC13039398; doi:10.1038/s41398-026-03981-z)
Supplement: Supplementary file 1 — Supplement Table [file 41398_2026_3981_MOESM1_ESM.docx]

Supplement Table 1. Parameters of Blue LED Panel

| Panel Dimensions | 310*270 mm |
| --- | --- |
| Effective Emission Area | 290*250 mm |
| LED Wavelength | 470 nm±10nm |
| LED Luminous Flux | 70-75 lm |
| LED Voltage | 3.2 V |
| Illuminance Range | 250-1200 lux |
| Illuminance Uniformity | ≥90% |

Supplement Table 2. Quality Analysis of RNA Sequencing

| **Sample** | **Raw reads** | **Clean reads** | **Error rate%** | **Q30 (%)** | **GC%** |
| --- | --- | --- | --- | --- | --- |
| Ctrl 1 | 50750260 | 49259190 | 0.0277 | 91.7 | 51.48 |
| Ctrl 2 | 53073914 | 51974218 | 0.0271 | 92.27 | 51.6 |
| Ctrl 3 | 56437526 | 54765280 | 0.0269 | 92.43 | 51.46 |
| SPS-S1 | 52837740 | 51159996 | 0.0275 | 91.89 | 51.52 |
| SPS-S2 | 78033394 | 76208388 | 0.0275 | 91.95 | 51.52 |
| SPS-S3 | 50781674 | 49402646 | 0.0271 | 92.24 | 51.51 |
| SPS-S + I-LT1 | 52217258 | 50944524 | 0.0271 | 92.25 | 51.7 |
| SPS-S + I-LT2 | 88741158 | 86449218 | 0.028 | 91.5 | 51.44 |
| SPS-S + I-LT3 | 49467306 | 48227510 | 0.0272 | 92.2 | 51.39 |

Supplement Table 3. Primer sequences of DEGs of RNA Sequencing

| Genes | Primer sequence |
| --- | --- |
| *Bhmt* | **F: AAATCCGCACAGTAGCCACA** |
|  | **R: AAGCCGGCATCAACACAAAC** |
| *Snap25* | **F: GGATGAGCAAGGCGAACAAC** |
|  | **R: TCCTGATTATTGCCCCAGGC** |
| *Apba2* | **F: AATCTGTGACCACTGCTGCTT** |
|  | **R: CAGGGAAGTTCACAGGGTCTC** |
| *Amph* | **F: CAAAAACGTGCAAAAGCGGC** |
|  | **R: CGCTGAAGTCTGGTACCCTC** |
| *Mat2b* | **F: GGCTGTGCTGCACTAGAGG** |
|  | **R: CAGTGGCACCGGTAATGAGA** |
| *Grm8* | **F: TACCCCITCTTCTGTGGCAAG** |
|  | **R: CGCTTTCCCTCGCATACCAT** |
| *Napb* | **F: GCAGGGAAAGAACGAGAAGC** |
|  | **R: TTGGCGGCTTGACAAAATGC** |
| *Syt6* | **F: GCCACTGGAGTTAGGATGGO** |
|  | **R: ATGGTGTCGGAGGTGGTTTC** |
| *Slc17a6* | **F: GGGGAGAGCGCAAATCTGTT** |
|  | **R: GACAACATGCCAACCTTGCT** |
| *Gapdh* | **F: ACCCACACTTCTCCATTTCC** |
|  | **R: TGGTTCCCAGGATAGGACTC** |

Supplement Table 4. Two-way Anova Results of Behavior Test.

| **Behavioral test** | **Dependent variable** | **F (df1, df2) – Time** | **P** | **F (df1, df2) – Treatment** | **P** | **F (df1, df2) – Interaction** | **P** | **Interpretation** |
| --- | --- | --- | --- | --- | --- | --- | --- | --- |
| OFT | Total distance | 1.94 (1, 95) | 0.167 | 8.47 (4, 95) | <0.0001 | 2.61 (4, 95) | 0.040 | Minor interaction; locomotion stable |
| OFT | Central zone entries | 20.96 (1, 95) | <0.0001 | 45.60 (4, 95) | <0.0001 | 11.29 (4, 95) | <0.0001 | Significant interaction |
| EPM | Open-arm entries | 62.65 (1, 95) | <0.0001 | 55.42 (4, 95) | <0.0001 | 13.45 (4, 95) | <0.0001 | Significant interaction |
| EPM | Time in open arms | 201.6 (1, 95) | <0.0001 | 140.0 (4, 95) | <0.0001 | 24.62 (4, 95) | <0.0001 | Significant interaction |
| EPM | Distance in open arms | 116.8 (1, 95) | <0.0001 | 52.63 (4, 95) | <0.0001 | 16.14 (4, 95) | <0.0001 | Significant interaction |
| Freezing test | Freezing ratio (%) | 356.8 (1, 95) | <0.0001 | 147.1 (4, 95) | <0.0001 | 39.77 (4, 95) | <0.0001 | Strong interaction;  fear extinction |
