# Supplementary material for: Preventing traumatic stress–induced behavioral abnormalities in rats with blue light phototherapy
Source: Transl Psychiatry. 2026 Mar 27;16:211. doi: 10.1038/s41398-026-03981-z (PMC13039398; doi:10.1038/s41398-026-03981-z)
Supplement: Supplementary file 2 — Supplement Figure [file 41398_2026_3981_MOESM2_ESM.pdf]

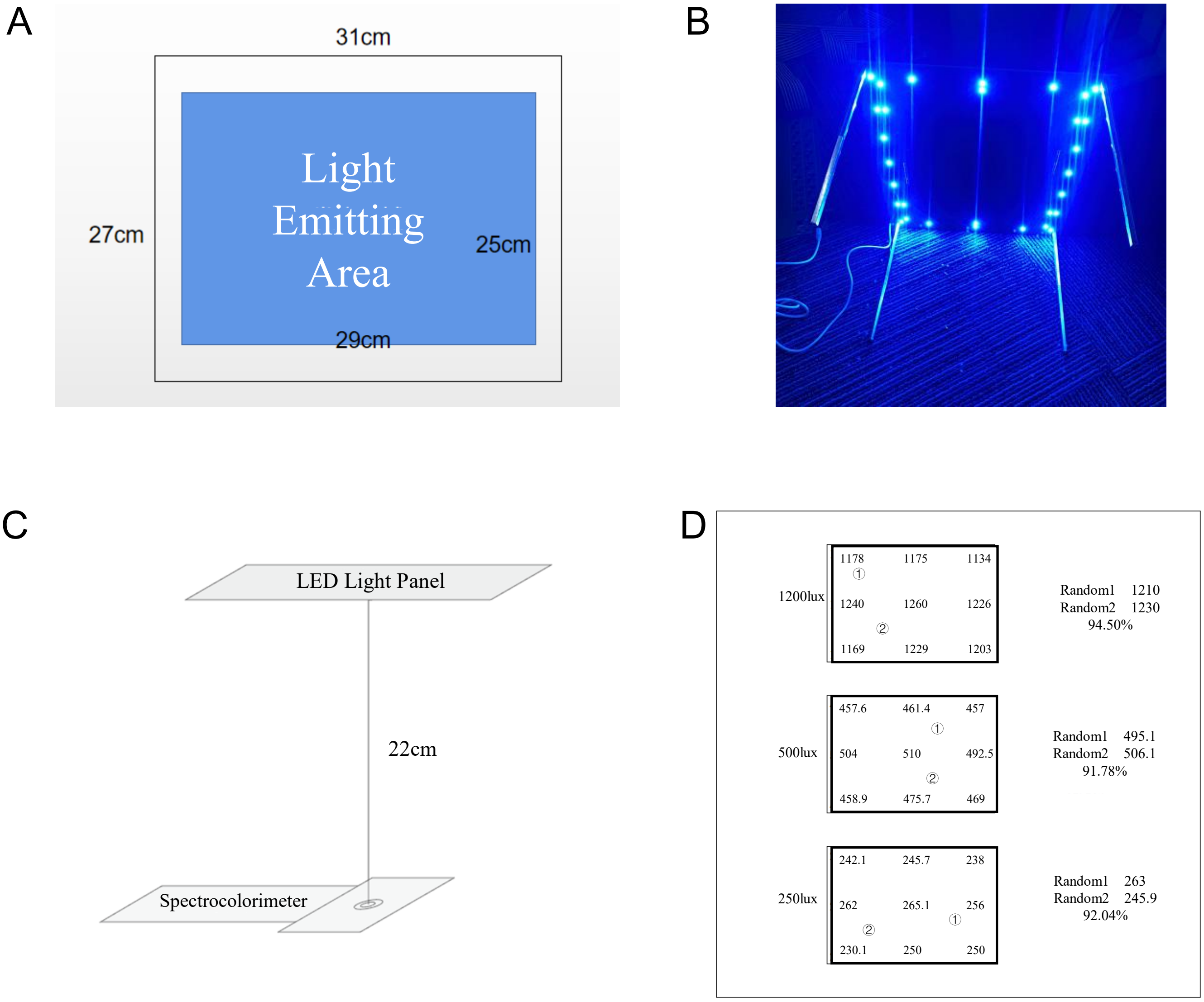

Supplement Figure 1. Schematic diagrams of the Light-Emitting Diode (LED) Light Panel. A. Diagram of the illuminated area of the light panel. B. Actual image of the light panel. C. Diagram of the illuminance measurement process of the light panel. D. Measurement results of illuminance at various points on the light panel.

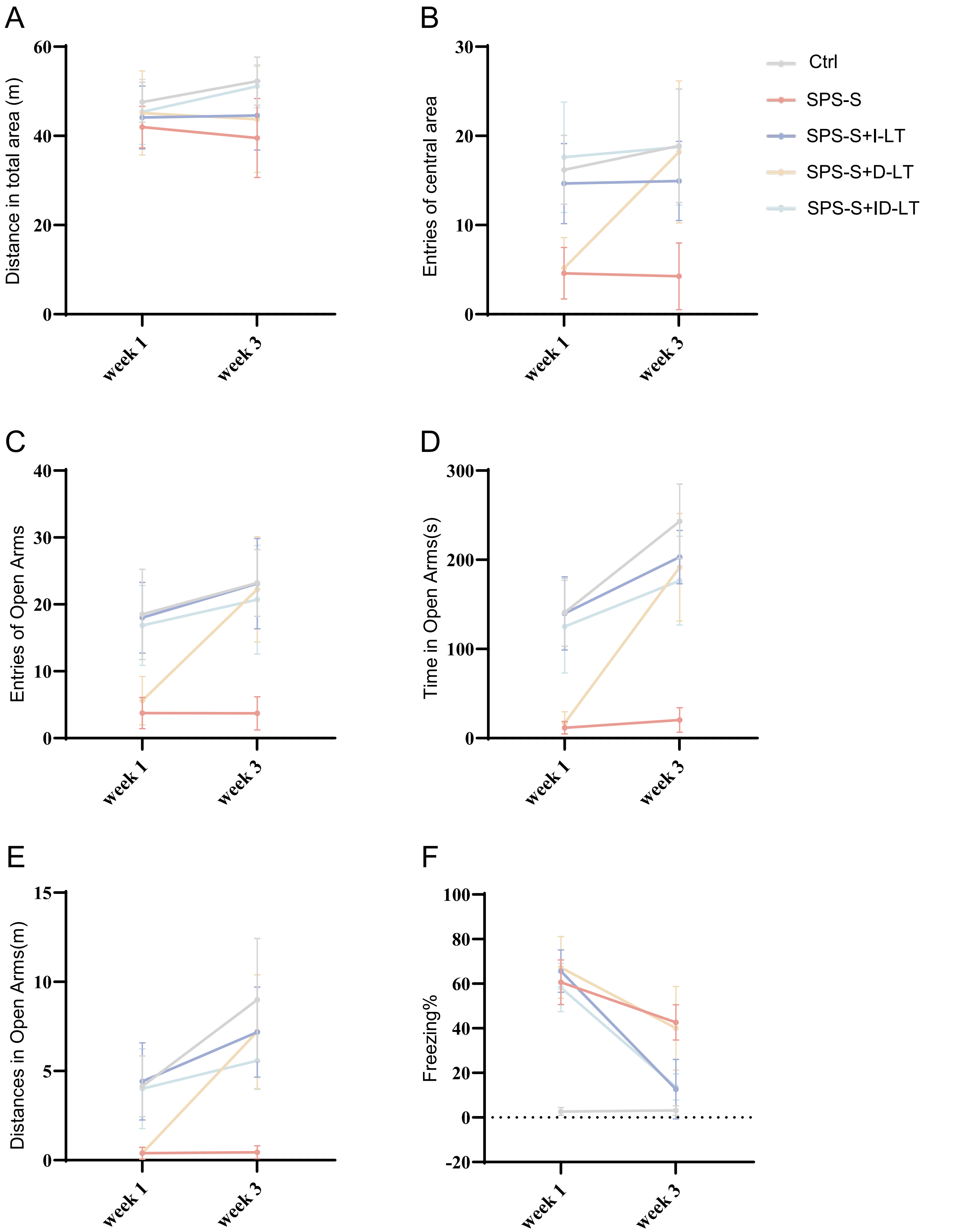

Supplement Figure 2. Two-way anova results of behavior test. A. Total distance moved in open field test (OFT). B. Entries into central area in OFT. C. Entries into Open Arms in elevated plus maze (EPM). D. Time spent in open arms in EPM. E. Distance moved in open arms in EPM. F. The percentage of freezing time in 6 mins.

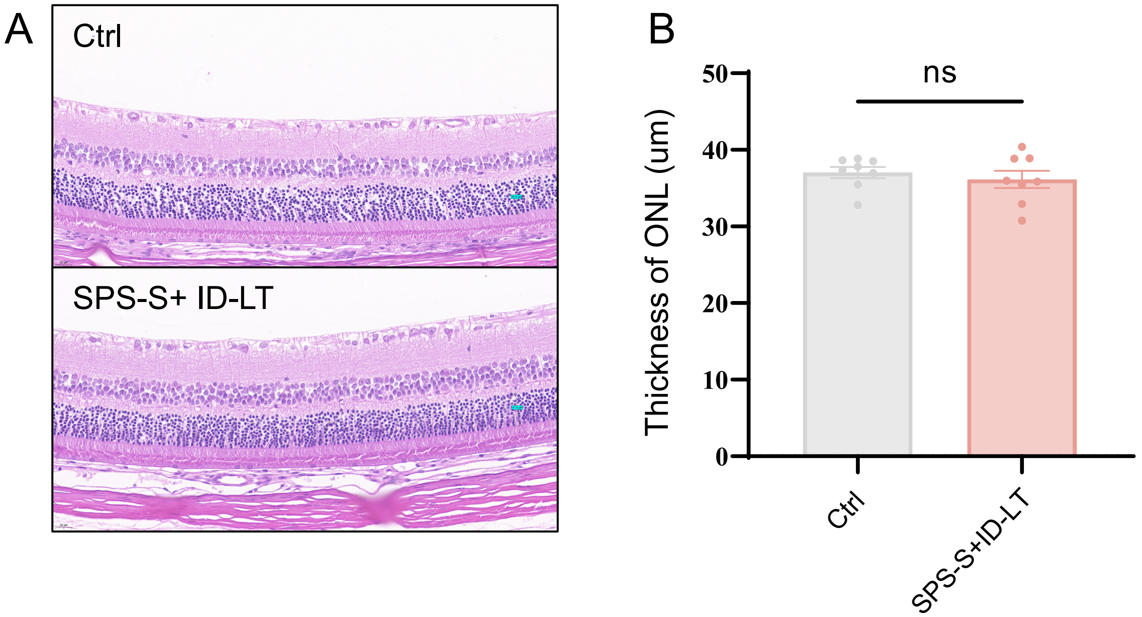

Supplement Figure 3. HE results of of Ctrl and ID-LT group. A. Hematoxylin–eosin (HE) staining schematic diagram. B. The thickness of outer nuclear layer (ONL). ns, no statistical difference. Each group, n = 8, t test.

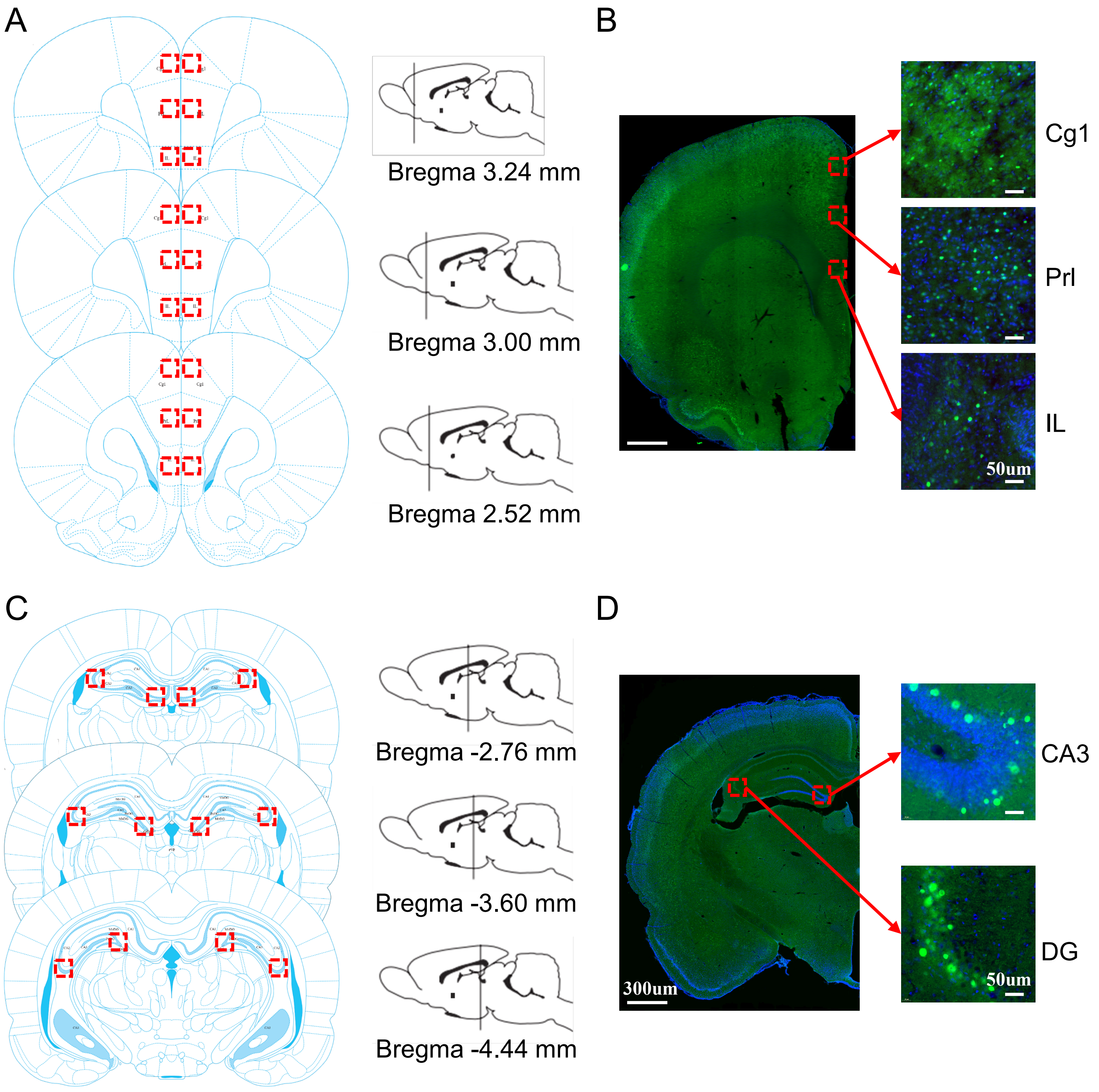

Supplement Figure 4. Schematic diagrams of immunofluorescence sections. A. Schematic diagram of medial prefrontal cortex (mPFC) section selection. B. Schematic diagram of mPFC subregion selection. C. Schematic diagram of hippocampal section selection. D. Schematic diagram of hippocampal subregion selection.

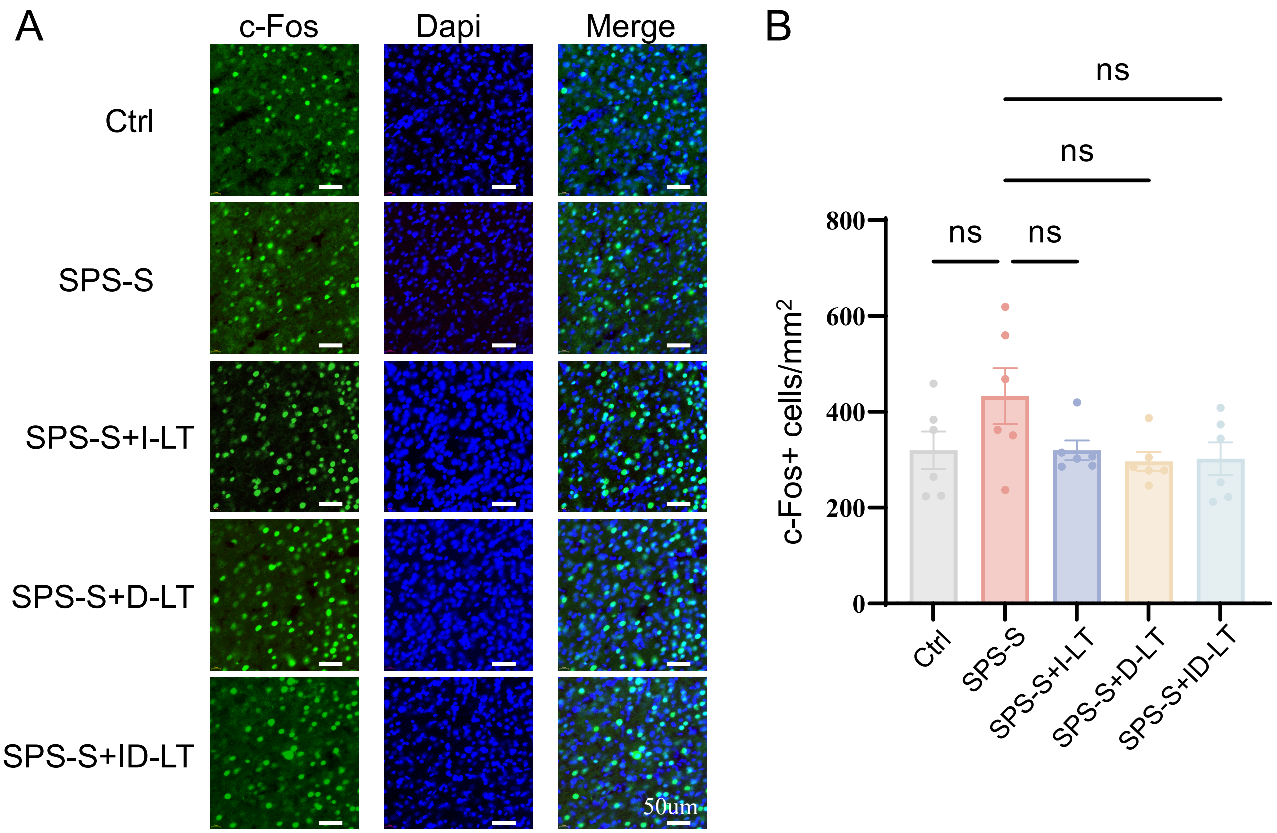

Supplement Figure 5. The effect of phototherapy on c-Fos expression in Cg1. A. Schematic diagram of immunofluorescence results in the Cg1 region. B. Statistical diagram of c-Fos density results in the Cg1 region. *P <0.05, **P <0.01, ***P <0.001, ns, no statistical difference. Each group, n = 6, one-way ANOVA.

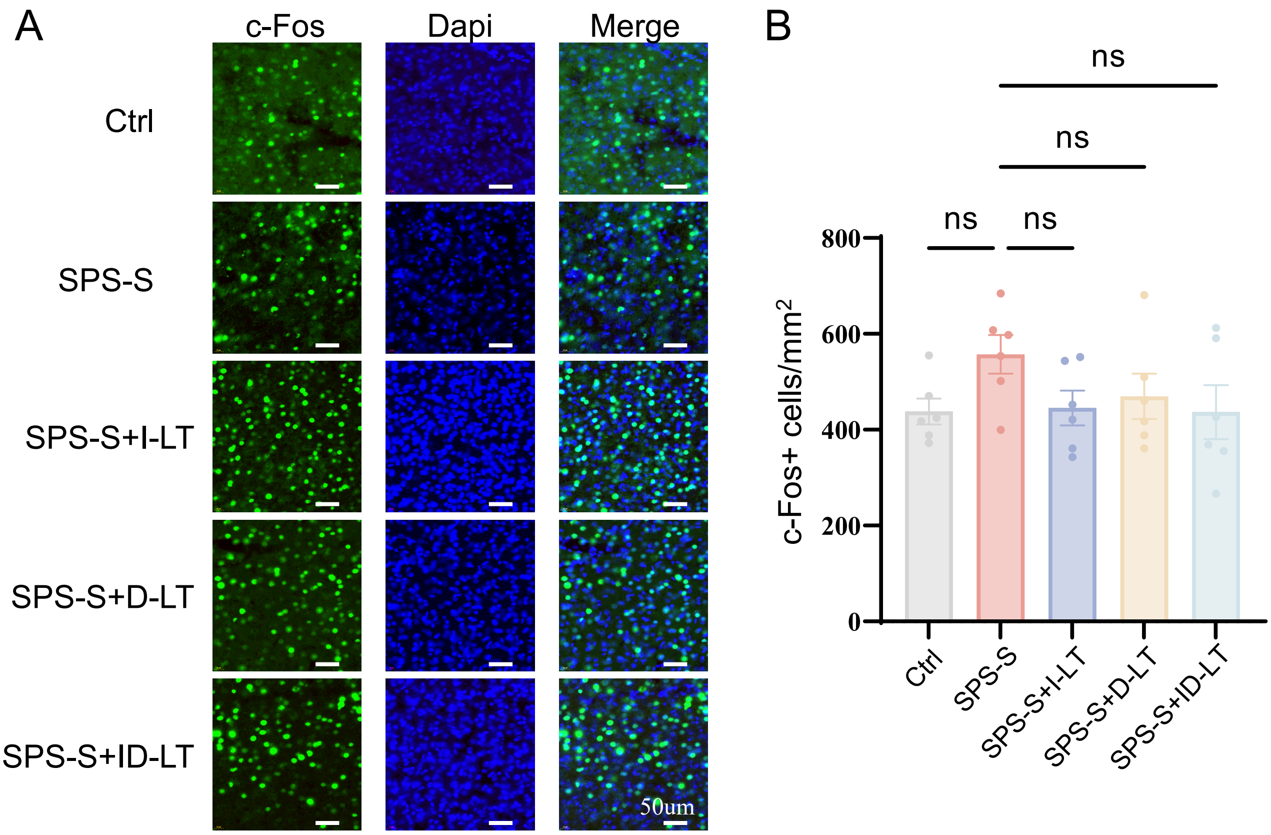

Supplement Figure 6. The effect of phototherapy on c-Fos expression in prelimbic cortex (Prl). A. Schematic diagram of immunofluorescence results in the Prl region. B. Statistical diagram of c-Fos density results in the Prl region. *P <0.05, **P <0.01, ***P <0.001, ns, no statistical difference. Each group, n = 6, one-way ANOVA.

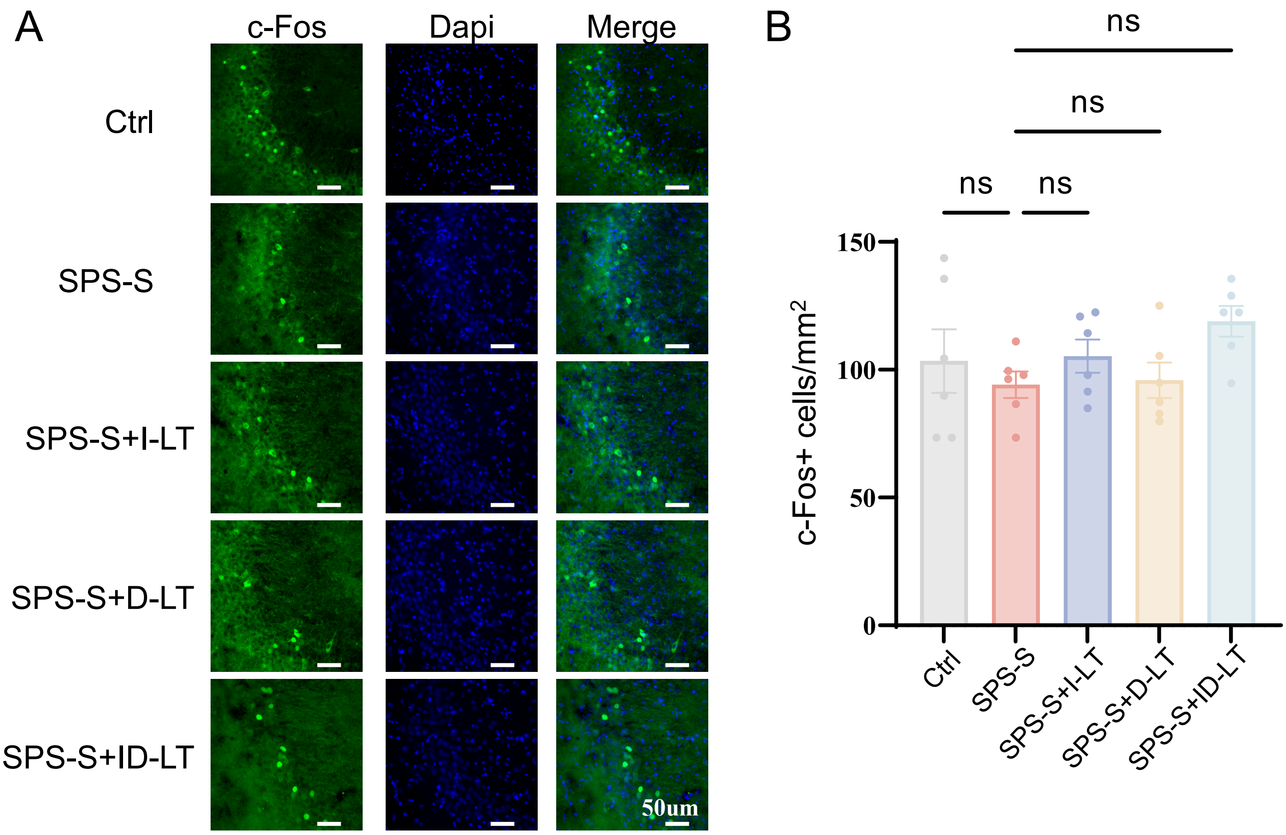

Supplement Figure 7. The effect of phototherapy on c-Fos expression in CA3. A. Schematic diagram of immunofluorescence results in the CA3 region. B. Statistical diagram of c-Fos density results in the CA3 region. *P <0.05, **P <0.01, ***P <0.001, ns, no statistical difference. Each group, n = 6, one-way ANOVA.

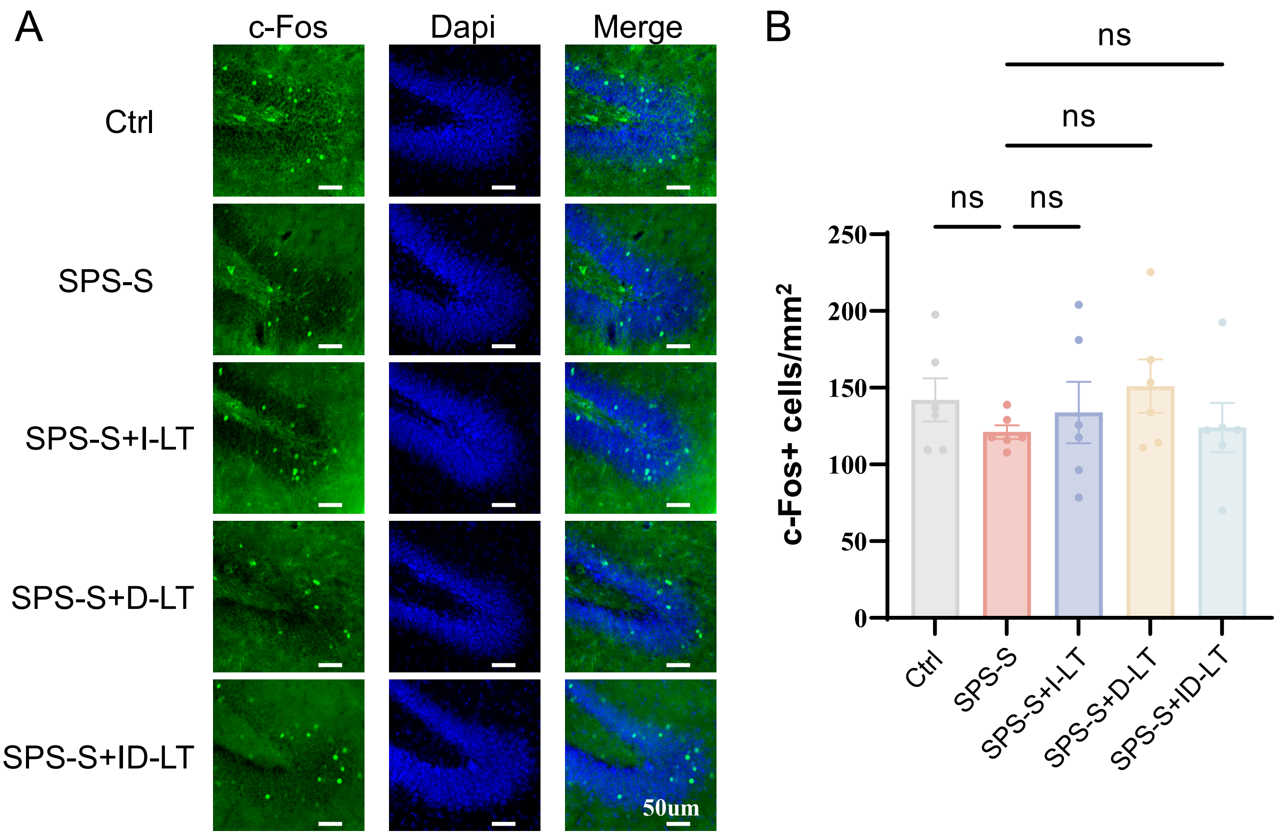

Supplement Figure 8. The effect of phototherapy on c-Fos expression in dentate gyrus (DG). A. Schematic diagram of immunofluorescence results in the DG region. B. Statistical diagram of c-Fos density results in the DG region. *P <0.05, **P <0.01, ***P <0.001, ns, no statistical difference. Each group, n = 6, one-way ANOVA.

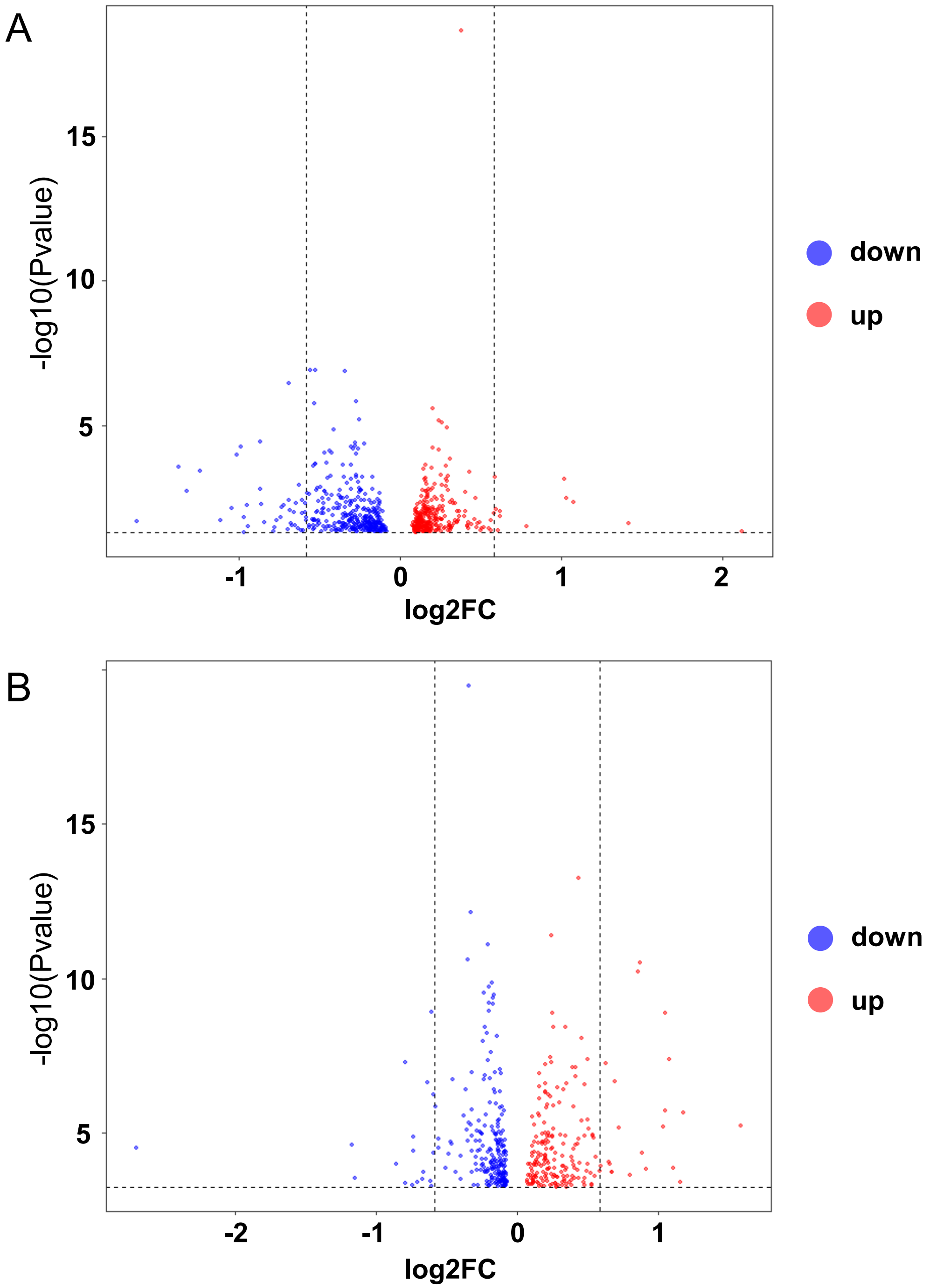

Supplement Figure 9. Volcano Plots of Differentially Expressed Genes (DEGs). A. Volcano plot of DEGs between the Ctrl and SPS-S groups. B. Volcano plot of DEGs between the SPS-S + I-LT and SPS-S groups. *DEGs were identified by RNA sequencing of tissue punches collected from the infralimbic mPFC (IL).

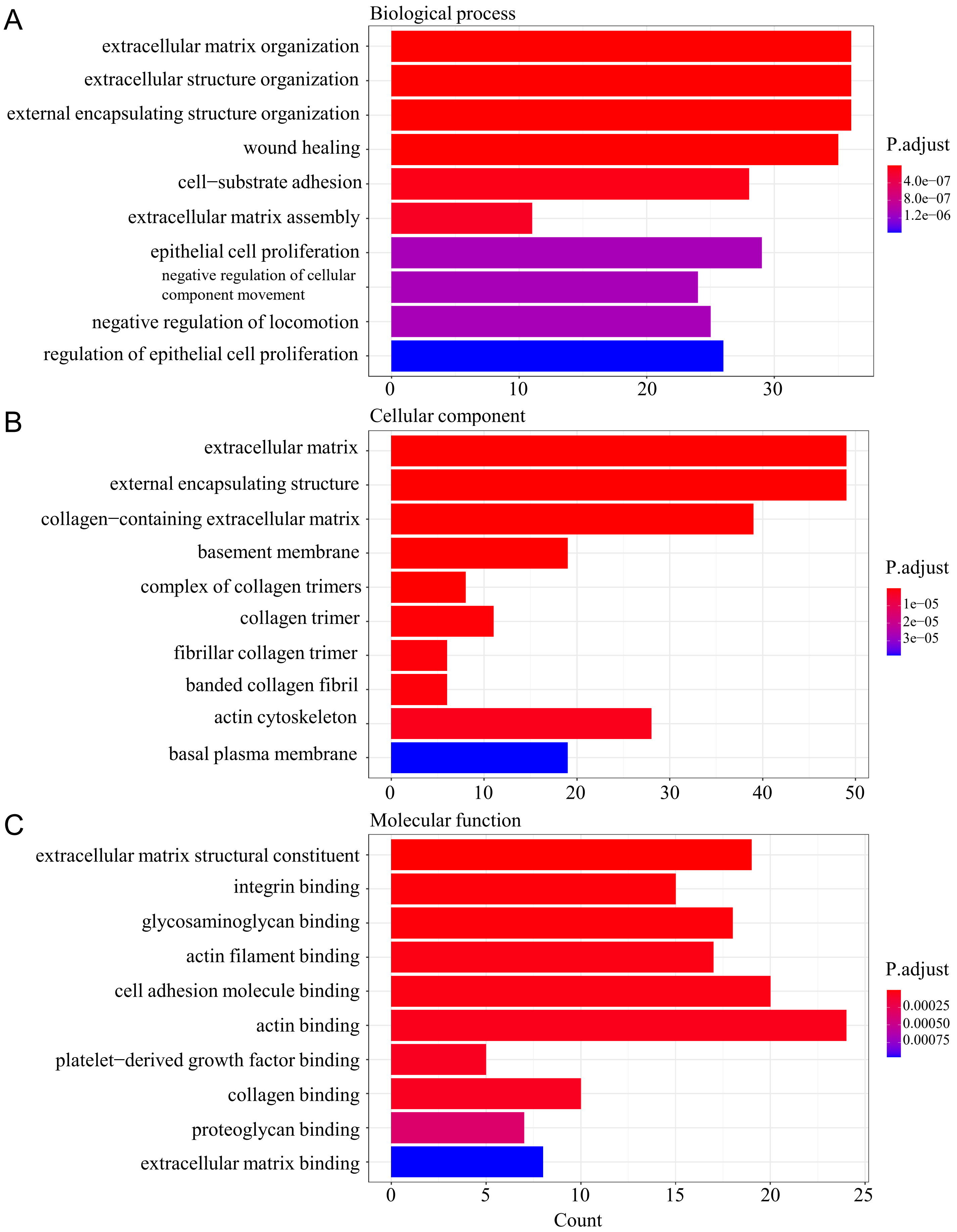

Supplement Figure 10. GO enrichment analysis of downregulated differentially expressed genes (DEGs) in the SPS-S group compared with the ctrl group. A. Top 10 enriched biological process pathways. B. Top 10 enriched cellular component pathways. C. Top 10 enriched molecular function pathways. *DEGs were identified by RNA sequencing of tissue punches collected from the infralimbic mPFC (IL).

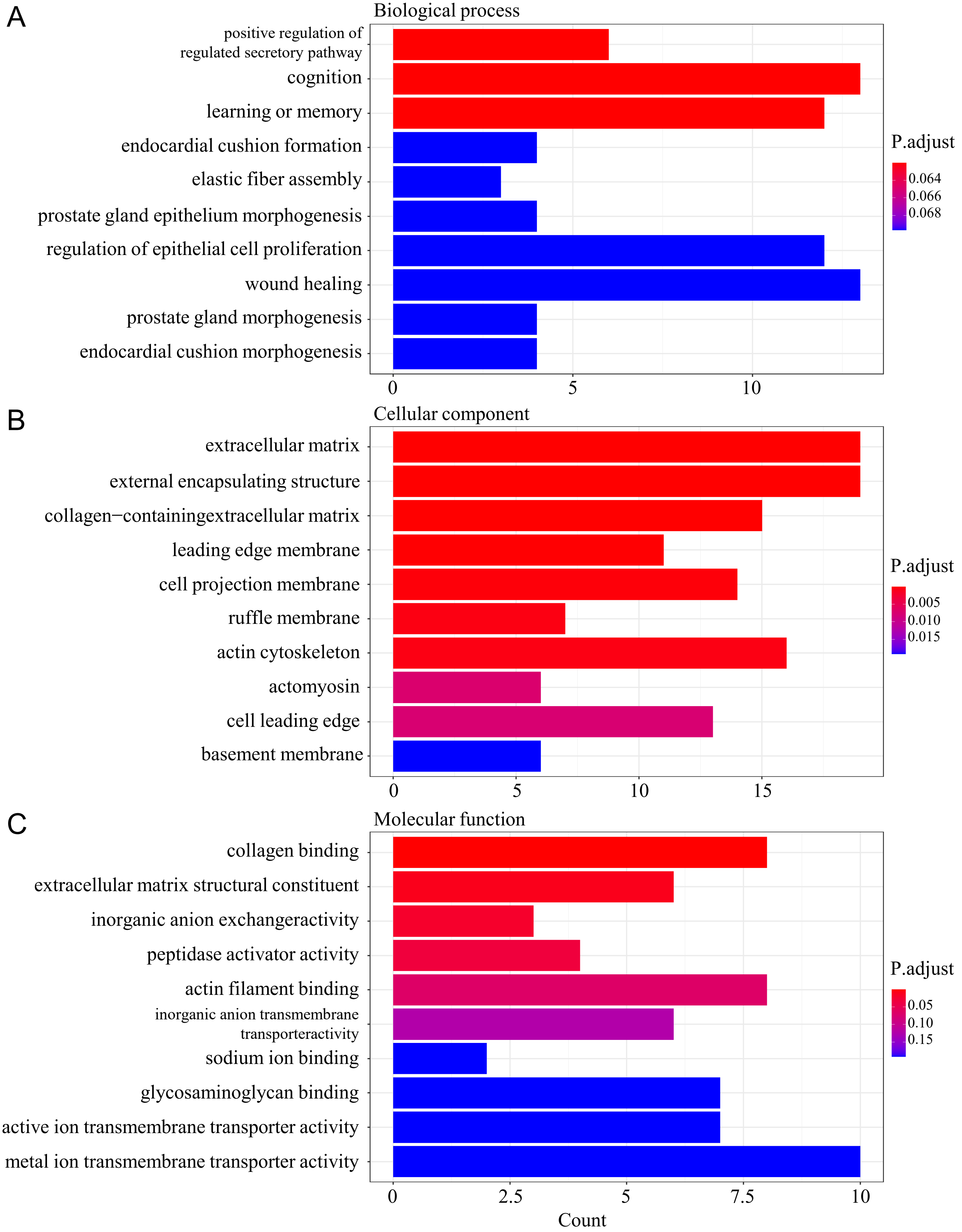

Supplement Figure 11. GO enrichment analysis of upregulated differentially expressed genes (DEGs) in the SPS-S+ I-LT group compared with the SPS-S group. A. Top 10 enriched biological process pathways. B. Top 10 enriched cellular component pathways. C. Top 10 enriched molecular function pathways. *DEGs were identified by RNA sequencing of tissue punches collected from the infralimbic mPFC (IL).

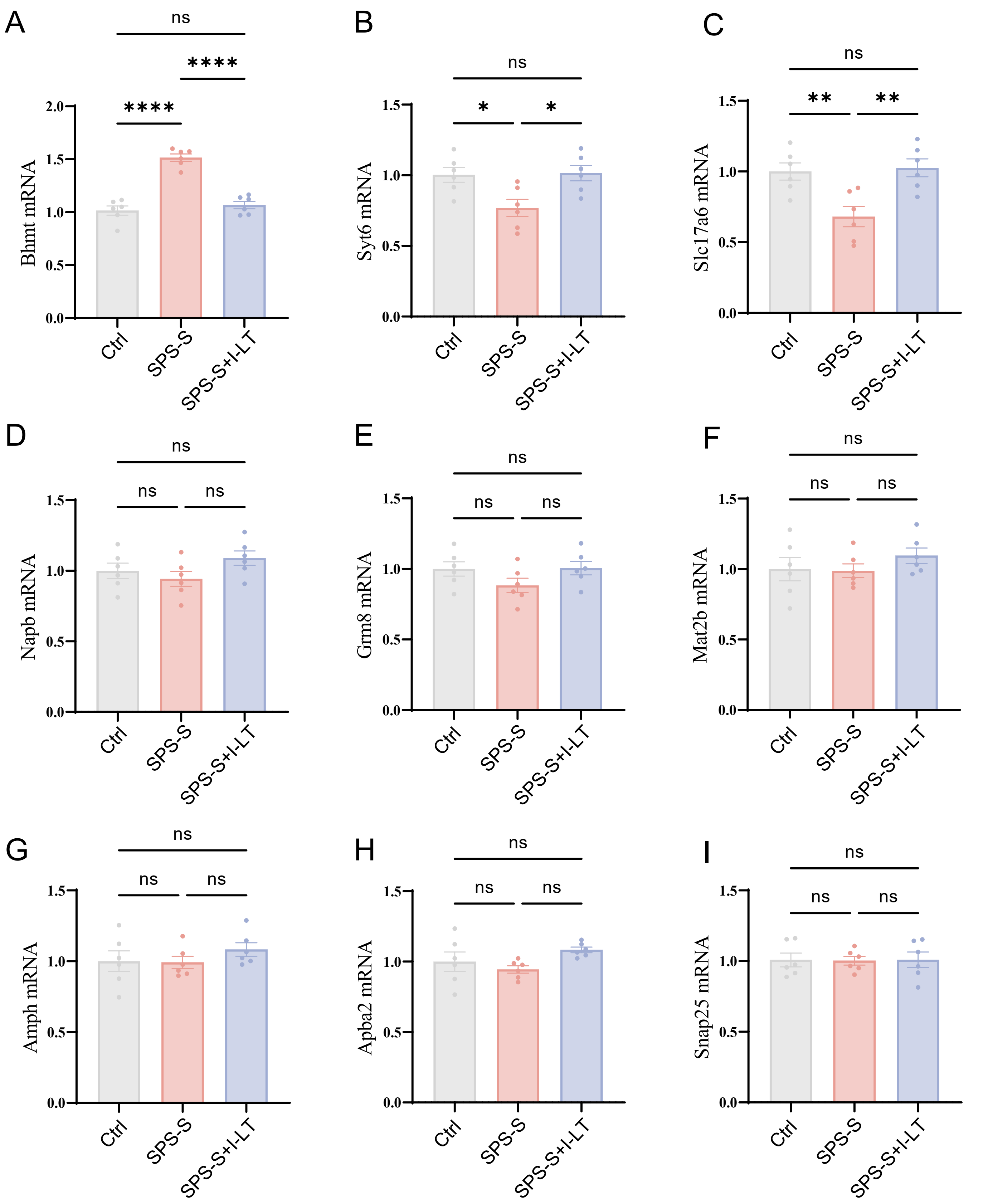

Supplement Figure 12. The relative mRNA expression levels of representative genes. A. The relative mRNA expression levels of Bhmt. B. The relative mRNA expression levels of Syt6. C. The relative mRNA expression levels of Slc17a6. D. The relative mRNA expression levels of Napb. E. The relative mRNA expression levels of Grm8. F. The relative mRNA expression levels of Mat2b. G. The relative mRNA expression levels of Amph. H. The relative mRNA expression levels of Apba2. I. The relative mRNA expression levels of Snap25. *P <0.05, **P <0.01, ***P <0.001, ns, no statistical difference. Each group, n = 6, one-way ANOVA.
